# Heart Failure and All-Cause Hospitalizations in Patients With Heart Failure: A Meta-Analysis

**DOI:** 10.1001/jamanetworkopen.2024.46684

**Published:** 2024-11-27

**Authors:** Ahmed Sayed, Mohamed ElRefaei, Kamal Awad, Husam Salah, John Mandrola, Andrew Foy

**Affiliations:** 1 Faculty of Medicine, Ain Shams University, Cairo, Egypt; 2 Houston Methodist DeBakey Heart & Vascular Center, Houston, Texas; 3 Faculty of Medicine, Zagazig University, Cairo, Egypt; 4 Division of Cardiology, Department of Medicine, Duke University, Durham, North Carolina; 5 Baptist Health, Louisville, Kentucky; 6 Division of Cardiology, Penn State Milton S. Hershey Medical Center, Hershey, Pennsylvania

## Abstract

**Importance:**

Heart failure (HF) hospitalization is a common end point in HF trials; however, how HF hospitalization is associated with all-cause hospitalization in terms of proportionality, correlation of treatment effects, and concomitant reporting has not been studied.

**Objective:**

To determine the ratio of HF to all-cause hospitalizations, whether reported treatment effects on HF hospitalization are associated with treatment effects on all-cause hospitalization, and how often all-cause hospitalization is reported alongside HF hospitalization.

**Data Sources:**

PubMed was searched from inception to September 2, 2024, for randomized clinical trials (RCTs) of HF treatments using MeSH (medical subject heading) terms and keywords associated with heart failure, ventricular failure, ventricular dysfunction, and cardiac failure, as well as the names of specific journals.

**Study Selection:**

RCTs of HF treatments and reporting on HF hospitalization published in 1 of 3 leading medical journals (*New England Journal of Medicine*, *The Lancet*, or *JAMA*).

**Data Extraction and Synthesis:**

The PRISMA guidelines were followed. Data extraction was performed by 2 reviewers, and disagreements were resolved by consensus. Trial baseline characteristics and outcome data on HF and all-cause hospitalizations were extracted. The ratio of HF to all-cause hospitalizations was calculated. The association of HF hospitalization effects with all-cause hospitalization effects was evaluated using hierarchical bayesian models with weak priors. The posterior distribution was used to calculate the HF hospitalization treatment effects that would need to be observed before a high probability (97.5%) of a reduction in all-cause hospitalization could be achieved. The proportion of trials reporting all-cause hospitalization was calculated.

**Main Outcomes and Measures:**

HF and all-cause hospitalizations.

**Results:**

Of 113 trials enrolling 261 068 patients (median proportion of female participants, 25.4% [IQR, 21.3%-34.2%]; median age, 66.2 [IQR, 62.8-70.0] years), 60 (53.1%) reported on all-cause hospitalization. The weighted median ratio of HF to all-cause hospitalizations was 45.9% (IQR, 30.7%-51.7%). This ratio was higher in trials with greater proportions of New York Heart Association class III or IV HF, with lower left ventricular ejection fractions, investigating nonpharmaceutical interventions, and that restricted recruitment to patients with HF and reduced ejection fraction. Reported effects on HF and all-cause hospitalizations were well-correlated (*R*^2^ = 90.1%; 95% credible interval, 62.3%-99.8%). In a large trial, the intervention would have to decrease the odds of HF hospitalization by 16% to ensure any reduction, 36% to ensure a 10% reduction, and 56% to ensure a 20% reduction in the odds of all-cause hospitalization with 97.5% probability.

**Conclusions and Relevance:**

In this meta-analysis of HF trials, all-cause hospitalization was underreported despite a large burden of non-HF hospitalizations. Large reductions in HF hospitalization must be observed before clinically relevant reductions in all-cause hospitalization can be inferred.

## Key Points

**Question:**

What is the association of heart failure (HF) hospitalization with all-cause hospitalization in terms of concomitant reporting, proportionality, and correlation of treatment effects?

**Findings:**

In this meta-analysis of 261 068 unique patients from 113 unique trials, nearly half the trials did not report all-cause hospitalization and, when reported, the ratio of HF to all-cause hospitalizations was typically just under half. Large reductions in HF hospitalization must be demonstrated before important reductions in all-cause hospitalizations can be inferred.

**Meaning:**

These findings suggest that a large proportion of hospitalizations in HF trials are not related to HF and that sizeable reductions in HF hospitalization are needed to ensure clinically relevant reductions in all-cause hospitalization.

## Teaser

This meta-analysis evaluates the ratio of heart failure (HF) to all-cause hospitalizations in randomized clinical trials, whether differences in all-cause hospitalization can be inferred from reported treatment effects on HF hospitalization, and how frequently these are reported.

## Introduction

In randomized clinical trials (RCTs) evaluating treatments for patients with heart failure (HF), HF hospitalization is a commonly used end point due to its clinical relevance and its ability to provide adequate statistical power. When the primary aim is to demonstrate the intervention’s disease-specific activity, it is an invaluable end point. However, patients with HF experience a substantial burden of hospitalizations unrelated to HF.^1^ In a patient population where multimorbidity is the norm rather than the exception^2^ and where hospitalizations occur due to many different causes,^1,3^ HF hospitalization may not necessarily account for most all-cause hospitalizations. Further, treatments that decrease HF hospitalization may have adverse effects that increase non-HF hospitalization and reduce all-cause hospitalization.

We therefore sought to answer 3 questions. (1) What is the ratio of HF to all-cause hospitalizations in HF RCTs? (2) Can treatment effects on all-cause hospitalization be inferred from treatment effects on HF hospitalization? If so, how large do reductions in HF hospitalization need to be to ensure a reduction in all-cause hospitalization? (3) How frequently are effects on all-cause hospitalization reported?

## Methods

### Search Strategy and Inclusion Criteria

Only RCTs enrolling patients with HF that reported on HF hospitalizations as an end point were eligible for inclusion. RCTs in the following 3 journals were eligible: the *New England Journal of Medicine*, the *Lancet*, and *JAMA*. These journals were chosen because RCTs published in them have the greatest impact on HF treatment guidelines, and trials in these journals generally have a higher methodological quality and a lower risk of bias.^4,5^

The search terms and strategy are shown in eMethods in Supplement 1. We searched PubMed because all 3 journals are indexed in it. PubMed was searched from inception to April 14, 2022. The search was updated 4 times: March 3, 2023; September 29, 2023; April 28, 2024; and September 2, 2024. We used MeSH (medical subject heading) terms and keywords associated with HF, ventricular failure, ventricular dysfunction, and cardiac failure, as well as the names of the 3 journals. All records were independently screened for eligibility by 2 reviewers (M.E. and K.A.), and their decisions were reviewed and confirmed by a third reviewer (A.S). The conduct of this work was guided by the Preferred Reporting Items for Systematic Reviews and Meta-analyses (PRISMA) guidelines. Because the study used publicly available data with no patient involvement, Ain Shams University Institutional Review Board deemed it exempt from institutional review board review and informed consent.

### Data Extraction

Baseline characteristics and outcome data were extracted into a standardized spreadsheet. Relevant data included the sample sizes of the intervention and control groups, follow-up durations, mean age, proportions of female participants, mean left ventricular ejection fraction (LVEF), the proportion of patients with New York Heart Association (NYHA) class III or IV HF enrolled, year of trial publication, and the number of participants hospitalized for HF and for any cause in each arm. To ensure that no data were missed, outcome data were extracted from the original publication, additional publications based on the original trial (regardless of the journal in which they were published), associated supplementary appendices, and results posted on ClinicalTrials.gov. Data extraction was performed by 2 reviewers (A.S. and K.A.), and disagreements were resolved by consensus.

### Outcomes of Interest

First, we quantified the ratio of patients who experienced HF hospitalization to those who experienced all-cause hospitalization. Second, we investigated the extent to which treatment effects on HF hospitalizations could adequately estimate treatment effects on all-cause hospitalizations. Third, we quantified the proportion of RCTs that reported on all-cause hospitalization.

### Statistical Analysis

#### Reporting of All-Cause Hospitalization

We calculated the overall proportion of RCTs reporting all-cause hospitalization. Using logistic regression models, we assessed the association between reporting all-cause hospitalization and trial characteristics that were associated with the ratio of HF to all-cause hospitalizations. We also reported the number of studies that had prespecified all-cause hospitalization as an outcome of interest in their protocols and whether they complied with its reporting. A trial was considered to have reported all-cause hospitalization regardless of whether this number was reported in the main text, the supplement, or associated publications and whether it was analyzed in the form of patients experiencing all-cause hospitalization or the total number of all-cause hospitalizations.

#### Ratio of All-Cause to HF Hospitalizations

The ratio of patients who experienced an HF hospitalization to those who experienced hospitalization due to any cause was calculated. Only the control arms of the eligible trials were included in this analysis to avoid distortion of our results by treatments that increase or decrease HF hospitalizations. We investigated whether this varied according to 6 trial characteristics: mean age (in years), the proportion of female participants, the proportion of patients with NYHA classes III and IV HF, mean LVEF, the type of intervention studied (pharmaceutical vs nonpharmaceutical), and whether the trial eligibility criteria were restricted to HF with reduced ejection fraction (HFrEF). This was performed using a linear model assuming a beta distribution for the observed ratio of HF to all-cause hospitalizations. Because beta distributions are naturally bounded between 0 and 1, they were a natural choice for our analysis, as the ratio of HF to all-cause hospitalizations is also bounded in this range (as the former is a subset of the latter). A logit link was used for the mean, and a maximum-likelihood approach was used for estimation. Effect sizes were reported as the interquartile percentage difference for continuous covariates (the difference between the ratios estimated at the first and third quartiles of the continuous covariate) and the between-group difference for binary covariates. These analyses were weighted by the inverse variance of the calculated ratio, such that trials with more precise estimates were more influential. To be eligible for this analysis, a trial must have reported on the raw number of patients experiencing HF and all-cause hospitalizations. Trials that did not report on the values of covariates were excluded from their respective analyses.

#### Assessment of Correlation Between All-Cause and HF Hospitalization Treatment Effects

Assessment of the correlation between all-cause and HF hospitalization treatment effects was performed using a bayesian hierarchical model with weak priors, allowing the data to dictate our posterior estimates. Our assessment was based on 3 factors. First, we calculated *R*^2^ values to establish how much of the variation in the treatment effect on all-cause hospitalization could be explained by their effect on HF hospitalization. Second, we estimated the slope of this correlation to determine how much of a reduction in all-cause hospitalization could be expected for a given reduction in HF hospitalization. Third, we calculated 95% posterior predictive intervals for a given reduction in HF hospitalization.

These intervals estimate a 95% probable range of treatment effects on all-cause hospitalization, given a specified treatment effect on HF hospitalization in a new trial. Two factors determine the width of this range. First is the extent to which the 2 treatment effects are correlated. The second is the uncertainty in estimating the treatment effect on HF hospitalization (such that a more precise estimate yields a narrower predictive interval). The uncertainty in HF hospitalization treatment effects is formally represented by their SE. Accordingly, we displayed posterior predictive intervals under 3 different scenarios in estimating the treatment effect on HF hospitalizations: no uncertainty in a hypothetical trial of infinite size (SE of 0), small uncertainty (mean SE of the most precise half of trials), and large uncertainty (mean SE of the least precise half of trials). The main analysis used odds ratios (ORs) due their favorable statistical properties, but 2 sensitivity analyses using risk ratios (RR) and absolute risk differences were additionally performed. Ratio-based measures (ORs and RRs) were analyzed on the log-scale and back-transformed to facilitate interpretability.

From these intervals, we calculated the minimum reduction in HF hospitalizations that would need to be observed for a new trial to have a 97.5% or higher probability of yielding any reduction in all-cause hospitalizations (OR or RR <1.0 and risk reduction <0), a small reduction (OR or RR <0.9 and risk reduction <1%), and a large reduction (OR or RR <0.8 and risk reduction <2%). The statistical rationale underlying these models can be found in the eMethods in Supplement 1. To be eligible for this analysis, a trial must have reported on the raw number of patients experiencing HF and all-cause hospitalizations.

All analyses were performed using R, version 4.2.0 (R Project for Statistical Computing).^6^ Bayesian modeling was performed using the brms package.^7^ Data visualization was performed using the ggplot2 package.^8^ For frequentist analyses, a 2-tailed *P* < .05 or a 95% CI excluding the null denoted statistical significance.

## Results

### Baseline Characteristics of Included Trials

eFigure 1 in Supplement 1 illustrates the study selection process. After screening a total of 707 records, we included 113 trials published between 1991 and 2024,^9,10,11,12,13,14,15,16,17,18,19,20,21,22,23,24,25,26,27,28,29,30,31,32,33,34,35,36,37,38,39,40,41,42,43,44,45,46,47,48,49,50,51,52,53,54,55,56,57,58,59,60,61,62,63,64,65,66,67,68,69,70,71,72,73,74,75,76,77,78,79,80,81,82,83,84,85,86,87,88,89,90,91,92,93,94,95,96,97,98,99,100,101,102,103,104,105,106,107,108,109,110,111,112,113,114,115,116,117,118,119,120,121^ of which 48 reported on the raw number of patients hospitalized for HF or any cause. The 113 trials enrolled a total of 261 068 patients with a median follow-up duration of 16.0 (IQR, 9.0-30.0) months. The median age of enrolled patients was 66.2 (IQR, 62.8-70.0) years, the median proportion of female participants was 25.4% (IQR, 21.3%-34.2%), the median proportion of male participants was 74.6% (IQR, 65.8%-7.8.7%), and the median proportion of participants with NYHA class III or IV HF was 52.0% (IQR, 32.1%-75.2%).

The median LVEF was 30.0% (IQR, 26.4%-33.1%). A total of 79 studies^10,11,12,13,15,16,17,18,19,20,21,22,23,24,25,26,27,28,29,30,31,32,33,35,37,38,41,42,43,44,45,46,49,51,52,53,54,55,56,57,61,62,65,67,68,69,70,71,72,73,74,75,76,77,78,80,81,82,83,84,85,86,87,88,90,91,94,96,100,101,102,103,106,109,110,113,117^ strictly recruited patients with HFrEF, whereas 34 studies^14,34,36,40,47,48,50,58,59,60,63,64,66,79,90,92,93,95,96,97,98,99,104,105,107,108,111,112,114,115,116,118,119,120,121^ recruited patients with HF with preserved or mildly reduced ejection fraction (HFpEF or HFmrEF) with or without HFrEF. When classified by the type of intervention studied, 71 of 113 trials^9,10,11,12,13,15,16,17,18,19,20,21,22,23,24,25,26,27,29,30,32,33,34,36,37,38,39,40,42,43,45,46,47,48,49,50,52,53,55,59,61,64,65,68,73,75,78,79,81,82,83,84,85,86,89,94,96,97,98,100,101,102,103,104,105,109,110,112,114,117,121^ studied pharmaceutical interventions and 42 trials^14,28,31,35,41,44,51,54,56,57,58,60,62,63,66,67,69,70,71,72,74,76,77,80,87,88,90,91,92,93,95,99,106,107,108,111,113,115,116,118,119,120^ studied nonpharmaceutical interventions. eTable 1 in Supplement 1 shows the baseline characteristics of all included trials, and eTable 2 in Supplement 1 shows the reasons for excluding other articles qualifying for full-text screening. eTable 3 in Supplement 1 shows the assessments using the Risk of Bias II tool for included studies.

### Reporting of All-Cause Hospitalization

**Figure 1.**
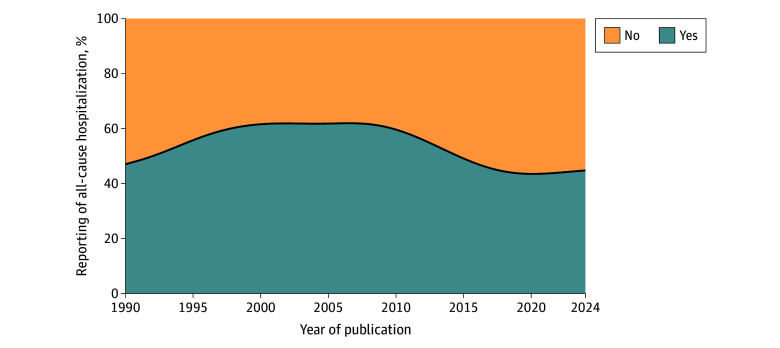
Proportion of Trials Reporting on All-Cause Hospitalization Across Time

Of the 113 trials included in this analysis, 60 (53.1%)^9,11,14,17,18,19,20,21,24,26,28,29,32,33,34,36,37,38,39,40,41,45,47,48,51,52,53,54,55,57,59,60,61,62,63,65,66,67,73,74,76,79,80,82,83,86,87,90,92,95,99,102,104,105,107,110,114,115,120,121^ reported on all-cause hospitalization, whereas 53 (46.9%)^10,12,13,15,16,22,23,25,27,30,31,35,42,43,44,46,49,50,56,59,64,68,69,70,71,72,75,77,78,81,84,85,88,89,91,93,94,96,97,98,100,101,103,106,108,109,111,112,113,116,117,118,121^ did not. The rates of reporting all-cause hospitalization did not improve across time (OR per 10-year increase, 0.79; 95% CI, 0.51-1.19) (Figure 1). There was no association between reporting all-cause hospitalization and the covariates that were associated with the ratio of HF to all-cause hospitalizations, including the proportion of patients with NYHA class III or IV HF (OR per 10 percentage points, 1.00; 95% CI, 0.86-1.15), LVEF (OR per 10 percentage points increase, 0.90; 95% CI, 0.57-1.40), class of intervention (OR for pharmaceutical vs nonpharmaceutical, 0.77; 95% CI, 0.35-1.66), and the enrollment of patients with HFrEF only (OR, 0.60; 95% CI, 0.26-1.36). Of the 99 trials with available preregistered protocols, 62 trials^9,11,12,16,19,23,25,30,32,35,36,42,43,44,45,46,50,56,59,63,64,66,68,69,70,71,72,73,74,75,76,77,81,83,84,85,86,89,91,93,94,95,96,97,100,101,102,103,104,105,106,108,109,111,112,113,116,117,118,121^ did not prespecify all-cause hospitalization as an end point of interest. Of 37 trials that prespecified all-cause hospitalization as an end point of interest, 35 trials^18,21,24,26,37,38,39,40,41,47,48,51,52,53,54,55,57,58,60,61,62,65,67,79,80,82,87,90,92,115,119,120^ reported on it.

### Ratio of HF Hospitalizations to All-Cause Hospitalizations

**Figure 2.**
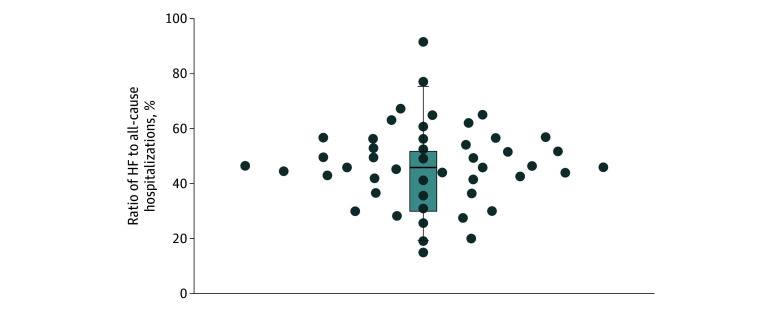
Ratio of Heart Failure (HF) to All-Cause Hospitalizations Across the Included Sample of Trials That Reported on the Number of Patients Experiencing Both Outcomes Circles represent individual studies (n = 47); the horizontal line in the box, the mean; and the whiskers, the 95% CIs.

**Figure 3.**
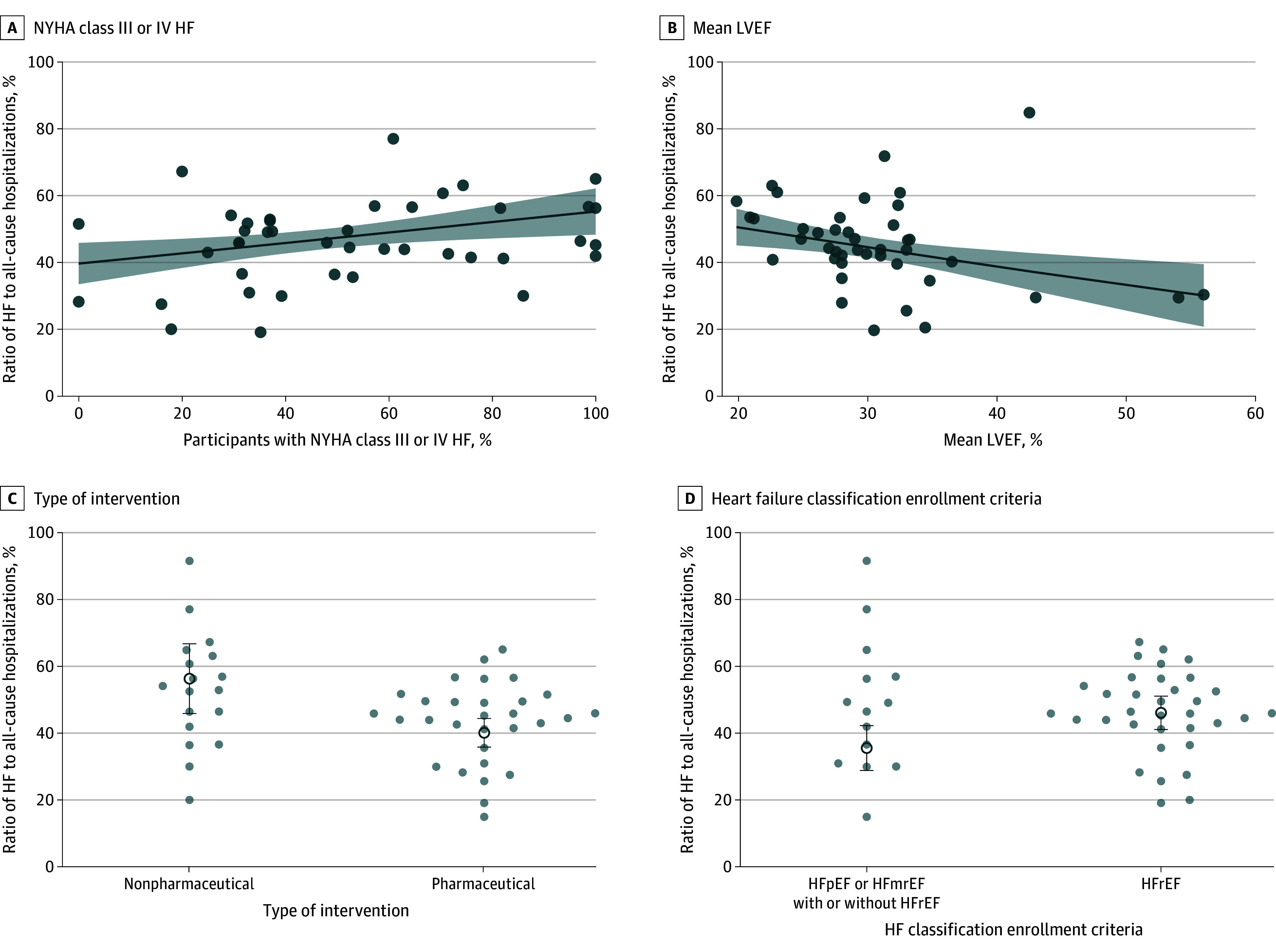
Differences in the Ratio of Heart Failure (HF) to All-Cause Hospitalizations A and B, Solid lines represent mean estimates; shading, 95% CIs; and circles individuals studies. C and D, Circles represent individual studies; error bars, 95% CIs around the mean. LVEF indicates left ventricular ejection fraction; mrEF, mildly reduced ejection fraction; NYHA, New York Heart Association; pEF, preserved ejection fraction; and rEF, reduced ejection fraction.

Among the control groups of the included trials, the weighted median ratio of HF to all-cause hospitalizations was 45.9% (IQR, 30.7%-51.7%) (Figure 2). This ratio increased in trials enrolling a greater proportion of participants with NYHA class III or IV HF (Figure 3A), with an interquartile percentage difference of 6.5% (95% CI, 1.5%-11.4%; *P* = .01). The ratio decreased in trials enrolling participants with higher mean LVEFs (Figure 3B), with an interquartile percentage difference of 3.5% (95% CI, 1.0%-6.0%; *P* = .01).

Additionally, the ratio was higher in trials of nonpharmaceutical interventions compared with pharmaceutical interventions (Figure 3C), with a percentage difference of 16.2% (95% CI, 4.9%-27.5%; *P* = .01). Finally, trials that restricted recruitment to patients with HFrEF had a higher ratio compared with trials that additionally enrolled patients with HFpEF or HFmrEF (Figure 3D), with a percentage difference of 10.5% (95% CI, 2.2%-18.9%; *P* = .02). There was no association between age and the proportion of female participants with this ratio.

### Assessment of the Association Between Treatment Effects on All-Cause and HF Hospitalization

**Figure 4.**
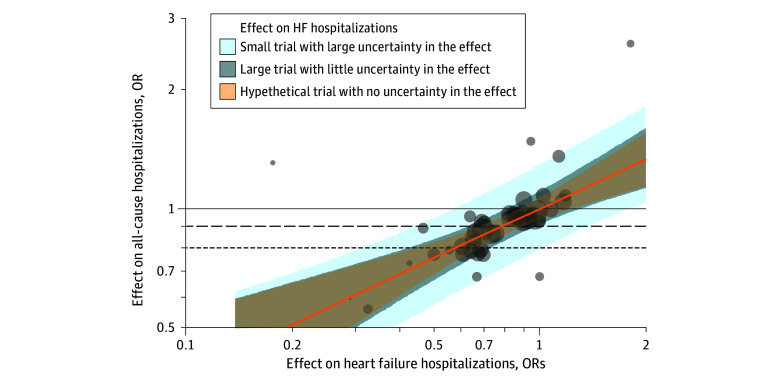
Correlation Between Treatment Effects on Heart Failure (HF) and All-Cause Hospitalizations Using Odds Ratios (ORs) The plot shows 3 predictive intervals (shading) under 3 scenarios: a hypothetical trial with no uncertainty in the effect on HF hospitalizations (light orange), a large trial with little uncertainty in the effect on HF hospitalizations (darker blue), and a small trial with large uncertainty in the effect on HF hospitalization (lighter blue). The horizontal black lines denote effects on all-cause hospitalizations for any reduction (solid; representing ≥0% odds reduction), a small reduction (dashed; representing ≥10% odds reduction), and a large reduction (dotted; representing ≥20% odds reduction). The orange line represents the regression line.

Figure 4 shows the value of HF hospitalization with respect to estimating all-cause hospitalization using the OR as a measure of effect size. Analyses using RRs and risk differences are shown in eFigures 2 and 3 in Supplement 1. First, reported treatment effects on HF and all-cause hospitalization were well-correlated (*R*^2^ = 90.1%; 95% credible interval [CrI], 62.3%-99.8%). The *R*^2^ values were 82.8% (95% CrI, 44.5%-99.4%) for RRs and 69.7% (95% CrI, 17.4%-98.5%) for absolute risk differences.

Second, the mean OR for all-cause hospitalization was approximately half that for HF hospitalization. Reductions of 25% and 50% in the odds of HF hospitalization would be expected to yield reductions of approximately 12.5% and 25%, respectively, in the odds of all-cause hospitalization (Figure 3). The corresponding values for RRs and risk differences can be seen in eFigures 2 and 3 in Supplement 1.

Third, a given treatment effect on HF hospitalization corresponded to a broad 95% predictive interval for all-cause hospitalization. For a hypothetical trial with no uncertainty in the treatment effect on HF hospitalization (corresponding to the shaded light orange interval in Figure 4), a 25% reduction in the odds of HF hospitalization could correspond to a reduction ranging from a 6% to a 16% reduction in the odds of all-cause hospitalization. Incorporation of the uncertainty in the treatment effect on HF hospitalizations (which is never reducible to 0 in real RCTs) resulted in a further widening of the predictive intervals, corresponding to the darker blue shaded interval (for a large trial with small uncertainty) and the lighter blue shaded interval (for a small trial with large uncertainty) in Figure 4.

The Table outlines the HF hospitalization reduction required to ensure a 97.5% predicted probability of the treatment reducing all-cause hospitalization by any amount, by a small amount (10% reduction in odds or risks or a 1% absolute risk reduction), and by a large amount (20% reduction in odds or risks or a 2% absolute risk reduction). For a large trial with small uncertainty in the estimated treatment effect on HF hospitalizations, the trialed treatment would need to reduce the odds of HF hospitalization by 16% to ensure any benefit in all-cause hospitalization, by 36% to ensure a small benefit, or by 56% to ensure a large benefit.

**Table.** Surrogate Threshold Effects for Heart Failure Hospitalization

Size of reduction in all-cause hospitalization	Effect size (95% CI)^a^		
	Odds ratio	Risk ratio	Absolute risk difference (percentage points)
**For a (hypothetical) infinitely sized trial with no uncertainty in estimating effect on HF hospitalizations**			
Required for a PPV of 97.5% for any reduction	0.87	0.89	−2.9
Required for a PPV of 97.5% for a small reduction^b^	0.67	0.47	−5.3
Required for a PPV of 97.5% for a large reduction^c^	0.45	0.18	−8.5
**For a large trial with minimal uncertainty in estimating effect on HF hospitalizations**			
Required for a PPV of 97.5% for any reduction	0.84 (0.74 to 0.95)	0.88 (0.83 to 0.93)	−3.5 (−5.8 to −1.2)
Required for a PPV of 97.5% for a small reduction^b^	0.64 (0.57 to 0.73)	0.47 (0.44 to 0.50)	−5.9 (−8.2 to −3.6)
Required for a PPV of 97.5% for a large reduction^c^	0.44 (0.39 to 0.50)	0.18 (0.16 to 0.19)	−8.9 (−11.2 to −6.6)
**For a small trial with high uncertainty in estimating effect on HF hospitalizations**			
Required for a PPV of 97.5% for any reduction	0.52 (0.28 to 0.97)	0.61 (0.37 to 1.00)	−8.6 (−17.8 to 0.6)
Required for a PPV of 97.5% for a small reduction^b^	0.41 (0.22 to 0.77)	0.35 (0.22 to 0.58)	−11.1 (−20.3 to −1.9)
Required for a PPV of 97.5% for a large reduction^c^	0.30 (0.16 to 0.55)	0.16 (0.10 to 0.26)	−14.1 (−23.3 to −4.9)

Abbreviations: HF, heart failure; PPV, positive predictive value.

^a^ Numbers inside parentheses denote the associated CIs for a trial with the corresponding required effect size and sample size. For the first category of hypothetical trials with infinite sample size, there is no uncertainty in estimating the effect on heart failure hospitalizations and therefore no CI.

^b^ A 10% reduction in odds or relative risks (risk or odds ratio of 0.9) or a 1% reduction in absolute risk. This can be deduced from Figure 3 (for odds ratios) and eFigures 2 and 3 in Supplement 1 (for risk ratios and risk reductions respectively) as the intersection between the intervals and the dashed black line.

^c^ A 20% reduction in odds or relative risks (risk or odds ratio of 0.8) or a 2% reduction in absolute risk. This can be deduced from Figure 3 (for odds ratios) and eFigures 2 and 3 in Supplement 1 (for risk ratios and risk reductions respectively) as the intersection between the intervals and the dotted black line.

## Discussion

HF hospitalization typically represents just under half of all hospitalizations in HF trials, though there is appreciable variability around this mean. This was greater in trials enrolling patients with a higher NYHA classification and lower LVEFs as well as trials investigating nonpharmaceutical interventions. Treatment effects on HF hospitalization are strongly associated with effects on all-cause hospitalization, though the HF hospitalization reductions necessary to ensure a high probability of achieving large reductions in all-cause hospitalization are beyond the reach of most interventions. Just under half of all trials reported on all-cause hospitalization, with no improvement across time.

The finding that HF hospitalizations typically account for half of the all-cause hospitalization burden in HF trials reflects the multimorbid nature of the disease, especially as HF trials are often enriched with patients who experience higher-than-average HF hospitalization rates. In a US community study,^122^ only 12.6% of all hospitalizations within 2 years of diagnosis were HF related and only 36% were related to a cardiovascular cause, substantially lower than the numbers reported herein.

Our assessment of the correlation between treatment effects on all-cause and HF hospitalizations shows 2 important findings. First, treatment effects on HF hospitalization were highly correlated with treatment effects on all-cause hospitalization, with *R*^2^ = 90.1% (though with a relatively large amount of uncertainty). For large trials, a 16% reduction in the odds of HF hospitalization would ensure a high probability of the treatment reducing all-cause hospitalization by any amount. However, to ensure a high probability of reducing all-cause hospitalizations by more than a modest amount (10% reduction in odds or greater), a 36% reduction would be necessary. Ensuring a high probability of a large reduction in all-cause hospitalization (20% reduction in odds or greater) required a reduction in the odds of HF hospitalizations by 56%, an unrealistic target for most interventions. For smaller trials, the necessary reductions are larger and likely unrealistic.

Although HF hospitalization itself may not be a surrogate outcome per se (as it has more relevance to patients than biomarkers or hemodynamic measures), using HF hospitalization to infer treatment effects on all-cause hospitalization is subject to the same principles governing the relationship between surrogate and true outcomes. The use of a surrogate outcome involves 3 main sources of uncertainty that exacerbate one another: (1) the uncertainty in the trial’s surrogate treatment effect; (2) the uncertainty in the relationship between effects on the surrogate outcome and the outcome of true interest; and (3) the uncertainty due to the residual variability that persists even after accounting for effects on the surrogate outcome. The first can be reduced by running larger trials (mitigating the cost and time savings of using a surrogate marker) whereas the second can be reduced by reporting treatment effects on all-cause hospitalization more frequently (so the association between the 2 outcomes can be more precisely estimated). The third is irreducible and would persist even if the exact effect on the surrogate outcome and the exact association between the surrogate and true outcomes were known with complete certainty.

The low overall level of all-cause hospitalization reporting observed—approximately 50%—poses problems for patients, clinicians, and regulators. For patients, it suggests that the data gained from their participation in clinical research is not being consistently used to its full extent. For clinicians, it is difficult to evaluate the full extent of the benefits and harms of a treatment if the data to do so are not available, as the lack of reporting makes it difficult to contextualize the relative importance of HF hospitalization and to consider the off-target harms (if any) that might lead to increased rates of non-HF hospitalization. For regulators, it is difficult to assess the net value of a treatment’s benefit if data on all-cause hospitalization are not available. Moreover, cost-effectiveness assessments, which should ideally assess an intervention in a holistic manner, are impeded by the lack of access to this data.

These problems are readily rectifiable by funding entities, regulatory agencies, and medical journals, all of which can enforce uniform reporting standards to increase reporting levels. Given the high levels of reporting among trials that prespecified all-cause hospitalization in their protocols, one solution may be for the US Food and Drug Administration to require the inclusion of this end point in trial protocols (even if it is not the primary outcome).

A helpful demonstration of the importance of reporting all-cause hospitalizations is provided by the discrepant relative effect of empagliflozin on all-cause hospitalizations in the EMPEROR-PRESERVED (Empagliflozin Outcome Trial in Patients With Chronic Heart Failure With Preserved Ejection Fraction)^104^ and EMPEROR-REDUCED (Empagliflozin Outcome Trial in Patients With Chronic Heart Failure With Reduced Ejection Fraction)^102^ trials (8% and 18% reductions, respectively), despite a similar HF hospitalization risk reduction in both trials (29% and 31%, respectively). This may be because the former trial, which enrolled patients with HFpEF, had a greater burden of non-HF causes of hospitalization (approximately 20% of hospitalizations were HF related). In contrast, the EMPEROR-REDUCED trial, which enrolled patients with HFrEF, had a greater burden of HF hospitalization (approximately 35% of hospitalizations were HF related), therefore allowing the similar reduction in HF hospitalization to translate to a substantially greater reduction in all-cause hospitalization.^102^ Other examples from the literature include the Digitalis Investigation Group trial, where digoxin reduced the risk of HF hospitalization by 28% and reduced the risk of all-cause hospitalization by only 8%, a 3.5-fold difference.^18^ This was partly due to a greater than 2 times increase in hospitalizations due to digoxin toxicity and a 1.2 times increase in other cardiovascular causes of hospitalization.^18^ In another example, a systematic review investigating brain natriuretic peptide guidance showed that this strategy reduced the risk of HF hospitalization (hazard ratio, 0.81; 95% CI, 0.68-0.98) but had little to no clear impact on all-cause hospitalization (hazard ratio, 0.97; 95% CI, 0.85-1.10).^123^

### Limitations

Several limitations of our meta-analysis merit caution. First, this was not an exhaustive sample of all HF trials. Trials in this analysis were selected from 3 leading medical journals because, as mentioned previously, these trials often have higher methodological quality and greater sample sizes and are more likely to inform guideline recommendations.^4,5^ Second, our assessment of the association between HF and all-cause hospitalization was based on 48 of the 113 included trials. Although this reflects low reporting rates, it also raises uncertainty as to what extent our findings may generalize to trials that withhold all-cause hospitalization effects (if this is due to selective rather than random omission). Moreover, data on all-cause hospitalization may sometimes not be published in the main report but in additional future publications. Although we attempted to obtain data from all retrievable sources, we cannot rule out that some trials included in our analysis will publish the all-cause hospitalization data at a future date. Further, some studies did not report covariate data necessary for our analysis of the ratio of HF to all-cause hospitalizations.

Further, the association between HF and all-cause hospitalizations may not be uniform in all settings because of differences in care for cardiac and noncardiac illnesses, differences in the prevalence of comorbidities, and differences in the illness thresholds at which patients are hospitalized. Finally, our models cannot account for future black swans (ie, an outlier treatment that has significant all-cause hospitalization benefit or harm not mediated by its treatment effect on HF hospitalization). In our sample, treatments that had no effect on HF hospitalization yielded, on average, no effect on all-cause hospitalization (as denoted by the regression line passing through the 1, 1 coordinate for ORs and RRs and the 0, 0 coordinate for absolute risk differences). There is no guarantee this average tendency will hold in the future or that significant but important deviations from this will not be observed. This statistical limitation further heightens the importance of uniformly reporting all-cause hospitalizations.

## Conclusions

In this meta-analysis of HF trials published in 3 leading medical journals, HF hospitalizations accounted for just under half of all-cause hospitalizations. This proportion was higher for trials enrolling more patients with NYHA class III or IV HF and lower LVEFs and in trials investigating nonpharmaceutical interventions. Although treatment effects on HF were highly correlated with treatment effects on all-cause hospitalizations, there was enough residual variability in the latter such that even large trials needed to demonstrate sizeable reductions in HF hospitalization before small reductions in all-cause hospitalization could be inferred with high certainty. For smaller trials, it may be impossible to infer reductions in all-cause hospitalization based on effects on HF hospitalization alone. Among HF trials that report HF hospitalizations, nearly 1 in 2 did not report on all-cause hospitalization. Given the importance of interpreting HF hospitalization within the broader context of all-cause hospitalization, these findings call for efforts to increase reporting rates.

## Notes

Supplement 1. **eMethods.** Search Strategy, Protocol, and Statistical Evaluation **eTable 1.** Characteristics of Included Studies **eTable 2.** Reasons for Excluding Articles That Qualified for Full-Text Screening **eTable 3.** Bias Assessments Across Included Studies **eFigure 1.** PRISMA Diagram Outlining the Study Selection Process **eFigure 2.** Correlation Between Treatment Effects on Heart Failure and All-Cause Hospitalization Using Risk Ratios **eFigure 3.** Correlation Between Treatment Effects on Heart Failure and All-Cause Hospitalization Using Absolute Risk Differences
eReferences.

Supplement 2.
Data Sharing Statement
