# Supplementary material for: Heart Failure and All-Cause Hospitalizations in Patients With Heart Failure: A Meta-Analysis
Source: JAMA Netw Open. 2024 Nov 27;7(11):e2446684. doi: 10.1001/jamanetworkopen.2024.46684 (PMC12549144; doi:10.1001/jamanetworkopen.2024.46684)
Supplement: Supplement 1. — eMethods. Search Strategy, Protocol, and Statistical Evaluation eTable 1. Characteristics of Included Studies eTable 2. Reasons for Excluding Articles That Qualified for Full-Text Screening eTable 3. Bias Assessments Across Included Studies eFigure 1. PRISMA Diagram Outlining the Study Selection Process eFigure 2. Correlation Between Treatment Effects on Heart Failure and All-Cause Hospitalization Using Risk Ratios eFigure 3. Correlation Between Treatment Effects on Heart Failure and All-Cause Hospitalization Using Absolute Risk Differences eReferences. [file jamanetwopen-e2446684-s001.pdf]

Supplementary Online Content

Sayed A, ElRefaei M, Awad K, Salah H, Mandrola J, Foy A. Heart failure and all-cause hospitalizations in patients with heart failure: a meta-analysis. *JAMA Netw Open*. 2024;7(11):e2446684. doi:10.1001/jamanetworkopen.2024.46684

eMethods. Search Strategy, Protocol, and Statistical Evaluation

eTable 1. Characteristics of Included Studies

eTable 2. Reasons for Excluding Articles That Qualified for Full-Text Screening

eTable 3. Bias Assessments Across Included Studies

eFigure 1. PRISMA Diagram Outlining the Study Selection Process

eFigure 2. Correlation Between Treatment Effects on Heart Failure and All-Cause Hospitalization Using Risk Ratios

eFigure 3. Correlation Between Treatment Effects on Heart Failure and All-Cause Hospitalization Using Absolute Risk Differences

eReferences.

This supplementary material has been provided by the authors to give readers additional information about their work.

© 2024 Sayed A et al. *JAMA Network Open*.

eMethods. Search Strategy, Protocol, and Statistical Evaluation

1. *Search Strategy*

We searched the MEDLINE/Pubmed database using the following search strategy: (((Heart failure[Title/Abstract] OR HF[Title/Abstract] OR HFrEF[Title/Abstract] OR HFpEF[Title/Abstract]) OR (Ventricular failure[Title/Abstract] OR ventricular dysfunction[Title/Abstract]) OR (CHF[Title/Abstract]) OR (Cardiac failure[Title/Abstract]) OR (heart failure[MeSH Terms])) AND ((randomized controlled trial[pt] OR controlled clinical trial[pt] OR randomized[tiab] OR placebo[tiab] OR clinical trials as topic[mesh:noexp] OR randomly[tiab] OR trial[ti] NOT (animals[mh] NOT humans [mh])))) AND (("The New England journal of medicine"[Journal]) OR ("JAMA"[Journal]) OR ("Lancet (London, England)"[Journal])). This was the only database used because all three journals (*New England Journal of Medicine, The Lancet, and JAMA)* included in this review were indexed on Pubmed.

2. *Protocol Registration*

Our assessment of surrogacy with respect to evaluating the relation between heart failure and all-cause hospitalization was registered on PROSPERO (CRD42022329505) within a broader evaluation of the relation of heart failure hospitalization to other clinical outcomes (namely mortality, all-cause hospitalization, and quality of life). The analyses with respect to mortality and quality of life are the subject of separate investigations outside the scope of this paper. The aims relating to the ratio of all-cause to HF hospitalizations and the frequency of reporting all-cause hospitalization were not pre-registered but were determined to be of interest during the conduct of the analysis.

3. *Additional details regarding the statistical evaluation of surrogacy*

Our analytic approach was primarily based on a previously established framework proposed for assessment of surrogacy in randomized clinical trials (RCTs).[114-116] There are 2 sequential stages to this approach. First, the effect of treatments on HF hospitalizations (the surrogate outcome) and all-cause hospitalizations (the outcome of interest) is estimated. The associated uncertainty (standard errors) of these estimates must also be quantified. Second, the correlation between effects on all-cause

© 2024 Sayed A et al. *JAMA Network Open*.

hospitalization (the outcome of interest) and effects on HF hospitalizations (the surrogate outcome) is estimated.

3.1 First stage: Estimation of treatment effects, standard errors, and intra-trial correlation coefficients in individual trials

In the first stage, we calculated the log odds ratio (log-OR), the log risk ratio (log-RR), and the absolute risk reduction (ARD) for each trial using the reported number of patients who experienced HF and all-cause hospitalizations in each treatment arm. The standard error of these treatment effects was estimated using a bootstrap sampling approach, wherein a random sample of patients in each trial is repeatedly selected and treatment effects calculated for each random sample. Subsequently, the standard deviation of treatment effects across these random samples is calculated and used as the estimate for their standard errors. In addition, the correlation between effects on HF and all-cause hospitalizations within a given trial is also calculated using the correlation estimate between this random sample of treatment effects. This should be accounted for as effects on HF and all-cause hospitalizations are expected to covary within the same trial (since a trial with a spuriously low or high treatment effect estimate on HF hospitalization is expected to yield a spuriously low or high treatment effect on all-cause hospitalizations). The first stage of modelling can be summarized as follows:

$$\begin{pmatrix} \hat{S}_i \\ \hat{T}_i \end{pmatrix} \sim \mathrm{N}\left(\begin{bmatrix} S_i \\ T_i \end{bmatrix}, \begin{bmatrix} \sigma^2_{S_i} & \sigma_{S_i}\sigma_{T_i}\rho_i \\ \sigma_{S_i}\sigma_{T_i}\rho_i & \sigma^2_{T_i} \end{bmatrix} \right)$$

In the above representation, the surrogate outcome (HF hospitalization) is represented using the letter *S* and the true outcome of interest (all-cause hospitalization) is represented using the letter *T*. $\hat{S}_i$ and $\hat{T}_i$ represent the observed estimate of the treatment effect on HF and all-cause hospitalization in trial *i*. This observed estimate is assumed to be drawn from a bivariate normal distribution. This bivariate normal distribution is defined by two mean parameters, S_i and T_i, which represent the actual (underlying)

© 2024 Sayed A et al. *JAMA Network Open*.

treatment effect on HF and all-cause hospitalization respectively. The observed estimate of HF and all-cause hospitalization treatment effects is subject to some uncertainty, defined by two parameters, σ_{Si} and σ_{Ti}, which represent the standard errors of these 2 respective outcomes. In addition, a correlation parameter, ρ_i, defines how the two observed treatment effect estimates are correlated within the same trial. This represents the previously alluded-to fact that spuriously high or low treatment effects on one outcome (HF hospitalization) are likely to be reflected as spuriously high or low treatment effects on another outcome (all-cause hospitalization). All of the 3 latter parameters (σ_{Ti}, σ_{Si}, and ρ_i) were estimated using bootstrap resampling. Although we did not have access to individual patient data, this approach was made possible by the fact that HF hospitalizations are nested (part of) all-cause hospitalizations, thereby allowing us to recreate datasets representing the outcomes observed in each trial.

3.2 Second stage: Estimation of between-trial correlation of treatment effects across the population of included trials

In the second stage of the model (estimating the correlation between treatment effects on HF and all-cause hospitalization), we estimated the average effect of treatments on HF and all-cause hospitalization across the present set of studies, the heterogeneity in effects on HF and all-cause hospitalization, and the correlation between effects on HF hospitalization and all-cause hospitalization. The second step of the model was performed using a Bayesian approach as it facilitates the propagation of uncertainty from the first modelling stage (namely, the uncertainty in the observed treatment effects) into the second modelling stage (namely, the uncertainty in the correlation between treatment effects). The second stage of modelling can be summarized as follows:

$$\begin{pmatrix} S_i \\ T_i \end{pmatrix} \sim \mathrm{N}\left(\begin{bmatrix} \mu_S \\ \mu_T \end{bmatrix}, \begin{bmatrix} \tau_S^2 & \tau_S \tau_T \rho \\ \tau_S \tau_T \rho & \tau_T^2 \end{bmatrix} \right)$$

© 2024 Sayed A et al. *JAMA Network Open*.

In the above representation, the (underlying) treatment effects on HF and all-cause hospitalization in a given trial (denoted by S_i and T_i respectively) are assumed to be drawn from a bivariate normal distribution. This bivariate normal distribution is represented by two mean parameters, μ_S and μ_T, representing the average effect of treatments on HF and all-cause hospitalization respectively among the population of RCTs. There is heterogeneity in the treatment effects among this population of RCTs, represented by τ_S and τ_T. The primary parameter of interest, ρ, represents the extent to which effects on HF and all-cause hospitalization are correlated across this population of RCTs.

3.3 Choice and rationale of priors

The Bayesian analysis used in the second modelling stage requires the specification of a set of priors for the different parameters in the given model. To ensure that our choice of priors did not materially impact our results, we used relatively weak and uninformative priors. For μ_S and μ_T, which represent the mean treatment effect across the included population of studies, we used a normal distribution with a mean of 0 and a standard deviation of 1 for analyses using log-odds ratios and log-risk ratios. This reflects a weak prior assumption that treatment effects are likely to lie (with 95% probability) between -2 and 2 on the log scale. When exponentiated to the natural scale, this corresponds to a weakly informative prior that treatment effects lie (with 95% probability) between odds/risk ratios of 0.14 to 7.4. For the analysis using absolute risk differences, we used a normal distribution with a mean of 0 and a standard deviation of 0.1. This corresponds to a weak prior assumption that treatment effects are likely to lie (with 95% probability) between a 20% decrease in the risk of hospitalization to a 20% increase in the risk of hospitalization. The width of these 95% intervals extends beyond what may be expected in nearly all major cardiovascular trials, meaning that they exert little to no effect on posterior estimates.

For heterogeneity in treatment effects, we used a half-Cauchy prior with a scale parameter of 1. This prior imposes little constrain on our estimate of heterogeneity and allows the observed variation in treatment effects in the dataset to dictate our posterior estimates of heterogeneity. For the correlation

© 2024 Sayed A et al. *JAMA Network Open*.

parameter, we used a Lewandowski-Kurowicka-Joe distribution assuming all correlations between -1 and 1 were equally likely.

© 2024 Sayed A et al. *JAMA Network Open*.

eTable 1. Characteristics of Included Studies

Trial (reference)	Year of publication	Type of intervention	Sample size (intervention group)	Sample size (control group)	Follow-up (months)	Age (intervention group; years)	Age (control group; years)	Females (intervention group; %)	Females (control group; %)	NYHA III/IV (intervention group; %)	NYHA III/IV (control group; %)	LVEF (intervention group; %)	LVEF (control group; %)
V-HeFT II (1)	1991	Drug	403	401	30.00	60.60	60.50	0.00	0.00	44.40	42.10	28.60	29.40
SOLVD-HF (B) (2)	1991	Drug	1,285	1,284	41.40	60.70	61.00	19.10	20.20	31.60	32.60	24.80	24.90
SAVE (3)	1992	Drug	1,115	1,116	36.00	59.30	59.50	17.00	18.00	NA	NA	31.00	31.00
SOLVD-HF (A) (4)	1992	Drug	2,111	2,117	37.40	59.10	59.10	11.50	11.40	0.00	0.00	28.00	28.00
Feldman et al., 1993 (5)	1993	Drug	239	238	6.00	58.10	58.20	13.40	13.00	78.60	82.40	20.00	20.00
Rich et al., 1995 (6)	1995	Strategy	142	140	3.00	80.10	78.40	68.00	59.00	NA	NA	44.00	41.00
PRAISE-1 (7)	1996	Drug	571	582	13.80	64.70	64.70	26.09	22.16	100.00	100.00	21.00	21.00
SWORD (8)	1996	Drug	1,549	1,572	27.00	60.40	59.90	14.00	14.00	22.00	21.00	31.00	30.80
ELITE (9)	1997	Drug	352	370	11.20	74.00	73.00	33.52	32.97	34.38	35.95	31.00	30.00
ANZ Trial (10)	1997	Drug	207	208	19.00	NA	NA	NA	NA	11.00	21.00	NA	NA
DIG trial (11)	1997	Drug	3,397	3,403	37.00	63.40	63.50	22.20	22.50	32.90	32.40	28.60	28.40
Cohn et al., 1998 (12)	1998	Drug	2,550	1,283	9.53	62.90	62.90	23.05	25.00	98.55	98.80	20.90	20.90
DIAMOND-CHF (13)	1999	Drug	762	756	18.00	70.00	70.00	28.00	25.00	62.00	58.00	NA	NA
CIBIS-II (14)	1999	Drug	1,327	1,320	15.60	61.00	61.00	19.00	20.00	100.00	100.00	27.50	27.60
RALES (15)	1999	Drug	822	841	24.00	65.00	65.00	27.00	27.00	99.50	99.60	25.60	25.20
IMPRESS (16)	2000	Drug	289	284	2.80	64.30	63.60	21.45	21.13	35.64	38.38	28.40	27.80
DIAMOND (17)	2000	Drug	749	761	15.20	68.00	69.00	28.00	25.00	35.00	36.00	NA	NA
ELITE II (18)	2000	Drug	1,578	1,574	18.50	71.40	71.50	30.00	31.00	48.00	48.00	31.00	31.00
MERIT-HF (19)	2000	Drug	1,990	2,001	12.00	63.90	63.70	23.00	22.00	59.40	58.80	28.00	28.00
Val-HeFT (20)	2001	Drug	2,511	2,499	23.00	62.40	63.00	20.10	20.00	37.80	38.50	26.60	26.90
BEST (21)	2001	Drug	1,354	1,354	24.00	60.00	60.00	21.00	23.00	100.00	100.00	23.00	23.00

© 2024 Sayed A et al. *JAMA Network Open*.

Trial (reference)	Year of publication	Type of intervention	Sample size (intervention group)	Sample size (control group)	Follow-up (months)	Age (intervention group; years)	Age (control group; years)	Females (intervention group; %)	Females (control group; %)	NYHA III/IV (intervention group; %)	NYHA III/IV (control group; %)	LVEF (intervention group; %)	LVEF (control group; %)
CAPRICORN (22)	2001	Drug	975	984	1.30	63.00	63.00	27.00	26.00	NA	NA	32.90	32.70
COPERNICUS (23)	2001	Drug	1,156	1,133	10.40	63.20	63.40	21.00	20.00	NA	NA	19.90	19.80
MUSTIC (24)	2001	Device	67	67	6.00	63.00	63.00	25.40	25.40	100.00	100.00	23.00	23.00
Troughton et al., 2000 (25)	2001	Strategy	33	36	9.70	68.00	72.00	22.00	25.00	28.00	33.00	28.00	26.00
MIRACLE (26)	2002	Device	228	225	6.00	63.90	64.70	32.00	32.00	100.00	100.00	21.80	21.60
OPTIMAAL (27)	2002	Drug	2,744	2,733	32.40	67.60	67.20	28.20	29.30	NA	NA	NA	NA
VMAC (28)	2002	Drug	273	216	6.00	62.00	60.00	27.00	40.00	86.00	76.00	NA	NA
CHARM Preserved (29)	2003	Drug	1,514	1,509	36.60	67.20	67.10	39.20	41.00	38.50	40.00	54.00	54.10
CHARM-Alternative (30)	2003	Drug	1,013	1,015	33.70	66.30	66.80	31.80	31.90	52.00	52.80	29.80	30.00
EPHESUS (31)	2003	Drug	3,319	3,313	16.00	64.00	64.00	28.00	30.00	NA	NA	33.00	33.00
COMPANION (32)	2004	Device	1,212	308	16.00	66.51	68.00	33.00	31.00	100.00	100.00	20.98	22.00
A-HeFT (33)	2004	Drug	518	532	10.00	56.70	56.90	44.20	36.10	99.80	100.00	23.90	24.20
ACTIV in CHF (34)	2004	Drug	239	80	2.00	62.00	60.00	31.38	25.00	94.98	93.80	24.00	25.00
CARE-HF (35)	2005	Device	409	404	29.40	67.00	66.00	26.00	27.00	100.00	100.00	25.00	25.00
CORONA (36)	2007	Drug	2,514	2,497	32.80	73.00	73.00	24.00	24.00	62.40	63.60	31.00	31.00
GISSI-HF (Fatty Acids) (37)	2008	Drug	3,494	3,481	46.80	67.00	67.00	22.20	21.20	36.30	36.80	33.00	33.20
GISSI-HF (Rosuvastatin) (38)	2008	Drug	2,285	2,289	3.90	68.00	68.00	23.80	21.40	38.80	36.10	33.40	33.10
ANDROMEDA (39)	2008	Drug	310	317	2.00	71.00	72.00	25.80	23.70	57.70	61.80	NA	NA
I-PRESERVE (40)	2008	Drug	2,067	2,061	49.50	72.00	72.00	59.00	61.00	80.00	79.00	59.00	60.00
BEAUTIFUL (41)	2008	Drug	5,479	5,438	12.00	65.30	65.00	17.00	17.00	24.00	23.00	32.40	32.30
ACCLAIM (42)	2008	Drug	1,204	1,204	10.20	64.60	64.00	20.00	20.00	70.00	73.00	22.70	22.60
AF-CHF (43)	2008	Rhythm Control	682	694	37.00	NA	NA	NA	NA	NA	NA	NA	NA

© 2024 Sayed A et al. *JAMA Network Open*.

Trial (reference)	Year of publication	Type of intervention	Sample size (intervention group)	Sample size (control group)	Follow-up (months)	Age (intervention group; years)	Age (control group; years)	Females (intervention group; %)	Females (control group; %)	NYHA III/IV (intervention group; %)	NYHA III/IV (control group; %)	LVEF (intervention group; %)	LVEF (control group; %)
TIME-CHF (44)	2009	Strategy	251	248	18.00	76.00	77.00	31.90	37.10	74.10	74.60	29.80	29.70
MADIT-CRT (45)	2009	Device	1,089	731	28.80	65.00	64.00	25.30	24.40	10.00	10.00	24.00	24.00
STITCH 2 (46)	2009	Surgery	501	499	48.00	62.00	62.00	14.00	16.00	49.00	50.00	28.00	28.00
HEAAL (47)	2009	Drug	1,921	1,913	56.40	66.00	66.00	30.00	29.00	31.00	31.00	33.00	33.00
FAIR-HF (48)	2009	Drug	304	155	5.60	67.80	67.40	52.30	54.80	82.60	81.30	31.90	33.00
RAFT (49)	2010	Device	894	904	40.00	66.10	66.20	15.20	19.00	20.80	19.20	22.60	22.60
SHIFT (50)	2010	Drug	3,241	3,264	22.90	60.70	60.10	24.00	23.00	52.00	52.00	29.00	29.00
PROTECT (51)	2010	Drug	1,356	677	0.23	70.20	70.20	32.70	33.20	NA	NA	32.30	32.50
HART (52)	2010	Strategy	451	451	31.17	63.80	63.40	46.30	48.30	31.90	31.30	NA	NA
Tele-HF (53)	2010	Strategy	826	827	6.00	61.00	61.00	43.50	40.60	57.80	56.70	NA	NA
ASCNED-HF (54)	2011	Drug	3,496	3,511	1.00	67.00	67.00	33.40	34.90	NA	NA	NA	NA
EMPHASIS HF (55)	2011	Drug	1,364	1,373	21.00	68.70	68.60	22.70	21.90	0.00	0.00	26.20	26.10
TIME (56)	2012	Intracoronary stem cells	79	41	6.00	56.78	57.00	12.60	12.20	NA	NA	36.30	37.30
FOCUS-CCTRN (57)	2012	Trans-endocardial stem cells	61	31	6.00	63.95	62.32	13.11	6.45	37.70	48.39	32.43	30.19
HF-ACTION (58)	2012	Lifestyle	1,158	1,164	30.00	59.00	59.00	30.00	30.00	38.00	36.00	25.00	25.00
CARRESS (59)	2012	Other	94	94	2.00	69.00	66.00	22.00	28.00	NA	NA	30.00	35.00
WARCEF (60)	2012	Drug	1,142	1,163	42.00	61.00	61.00	20.70	19.30	32.20	29.60	25.00	25.00
BLOCK HF (61)	2013	Device	349	342	37.00	73.70	73.00	23.20	27.20	26.90	27.80	40.30	39.60
EchoCRT (62)	2013	Device	404	405	19.40	57.60	58.30	27.20	28.10	97.80	96.30	27.00	27.00
ASTRONAUT (63)	2013	Drug	808	807	11.30	64.70	64.50	21.20	24.40	63.00	66.00	27.90	27.80
CELLWAVE (64)	2013	Intracoronary Ssem cells	43	39	4.00	61.00	61.50	20.30	15.10	37.30	28.40	NA	NA
CHARM-Added (65)	2003	Drug	1,276	1,272	41.00	64.00	64.10	21.20	21.40	75.60	76.20	28.00	28.00
RED-HF (66)	2013	Drug	1,136	1,142	28.00	72.00	71.00	40.30	42.60	67.40	63.00	31.00	30.00

© 2024 Sayed A et al. *JAMA Network Open*.

Trial (reference)	Year of publication	Type of intervention	Sample size (intervention group)	Sample size (control group)	Follow-up (months)	Age (intervention group; years)	Age (control group; years)	Females (intervention group; %)	Females (control group; %)	NYHA III/IV (intervention group; %)	NYHA III/IV (control group; %)	LVEF (intervention group; %)	LVEF (control group; %)
PARADIGM-HF (67)	2014	Drug	4,187	4,212	27.00	63.80	63.80	21.00	22.60	23.90	25.50	29.60	29.40
IN-TIME (68)	2014	Strategy	333	331	12.00	65.30	65.80	17.70	20.80	54.80	59.20	26.00	26.00
TOPCAT (69)	2014	Drug	1,722	1,723	39.60	68.70	68.70	51.60	51.50	33.40	32.60	56.00	56.00
TAC-HFT (70)	2014	Trans-endocardial stem cells	38	21	12.00	59.10	60.60	7.90	4.80	15.80	21.70	35.80	32.00
SERVE-HF (71)	2015	Device	666	659	31.00	69.60	69.30	10.10	9.10	70.60	70.30	32.50	32.20
SOCRATES (72)	2015	Drug	364	92	2.80	68.00	67.00	19.53	20.70	48.92	41.30	29.88	28.60
ATMOSPHERE (73)	2016	Drug	4,680	2,336	36.60	63.25	63.30	21.90	21.40	35.95	38.30	28.45	28.30
CUPID 2 (74)	2016	Drug	123	127	17.50	60.30	58.40	17.00	20.00	81.00	83.00	23.00	24.00
COSMIC-HF (75)	2016	Drug	150	149	4.70	63.00	64.00	15.50	20.00	32.00	30.00	29.30	29.15
STICHES (76)	2016	Surgery	610	602	117.60	60.00	59.00	73.00	75.00	37.00	37.00	27.00	28.00
MOOD-HF (77)	2016	Drug	185	187	18.60	62.20	62.30	24.00	25.00	48.00	58.00	34.90	34.70
FIGHT (78)	2016	Drug	154	146	5.97	62.00	61.00	20.00	23.00	65.00	70.00	25.00	25.00
GUIDE-IT (79)	2017	Strategy	446	448	15.00	62.00	64.00	31.00	33.00	42.00	43.00	24.00	25.00
TRUE-AHF (80)	2017	Drug	1,088	1,069	15.00	68.70	68.30	34.40	34.00	62.60	62.10	NA	NA
CASTLE-AF (81)	2018	Ablation	179	184	37.80	64.00	64.00	13.00	16.00	31.00	28.00	32.50	31.50
CULPRIT-SHOCK (82)	2018	Revascularization	344	342	12.00	70.00	70.00	25.10	21.90	NA	NA	33.00	30.00
MITRA FR (83)	2018	Valvular repair	152	152	12.00	70.10	70.60	21.10	29.60	63.10	71.10	33.30	32.90
COMMANDER HF (84)	2018	Drug	2,507	2,515	21.10	66.50	66.30	22.00	23.80	52.00	53.70	35.00	34.00
COAPT (85)	2018	Valvular repair	302	312	22.70	71.70	72.80	33.40	38.50	57.00	64.60	31.30	31.30
PIONEER-HF (86)	2019	Drug	440	441	2.00	61.00	63.00	25.70	30.20	73.20	69.20	24.00	25.00
PARAGON-HF (87)	2019	Drug	2,407	2,389	35.00	72.70	72.80	51.60	51.80	0.40	0.50	57.60	57.50
DAPA-HF (88)	2019	Drug	2,373	2,371	18.20	66.20	66.50	23.80	23.00	31.50	31.70	31.20	30.90
GALACTIC (89)	2019	Strategy	382	399	6.00	78.00	77.00	37.00	37.00	100.00	100.00	36.00	37.00

© 2024 Sayed A et al. *JAMA Network Open*.

Trial (reference)	Year of publication	Type of intervention	Sample size (intervention group)	Sample size (control group)	Follow-up (months)	Age (intervention group; years)	Age (control group; years)	Females (intervention group; %)	Females (control group; %)	NYHA III/IV (intervention group; %)	NYHA III/IV (control group; %)	LVEF (intervention group; %)	LVEF (control group; %)
PANACHE (90)	2019	Drug	229	76	4.67	73.10	74.00	54.10	47.00	12.40	15.00	55.70	57.00
PACT-HF (91)	2019	Strategy	1,104	1,390	3.00	77.77	77.59	49.30	51.40	NA	NA	NA	NA
EMPEROR-REDUCED (92)	2020	Drug	1,863	1,867	16.00	67.20	66.50	23.50	24.40	24.90	25.00	27.70	27.20
AFFIRM-AHF (93)	2020	Drug	558	550	12.00	71.20	70.90	44.00	45.00	52.00	54.00	32.60	32.70
VICTORIA (94)	2020	Drug	2,526	2,524	10.80	67.50	67.20	24.00	23.90	41.40	40.60	29.00	28.80
GALACTIC-HF (95)	2021	Drug	4,120	4,112	21.80	64.50	64.50	21.20	21.30	46.70	47.10	26.60	26.50
CONNECT-HF (96)	2021	Strategy	2,675	2,972	10.28	62.30	62.90	32.80	33.80	NA	NA	NA	NA
EMPEROR-Preserved (97)	2021	Drug	2,997	2,991	26.20	71.80	71.90	44.60	44.70	18.70	18.10	54.30	54.30
GUIDE-HF (98)	2021	Strategy (and device)	497	503	12.00	71.00	70.00	38.00	37.00	71.00	70.00	38.00	40.00
REHAB-HF (99)	2021	Lifestyle	175	174	6.00	73.10	72.20	49.00	56.00	80.00	81.00	NA	NA
SOLOIST-WHF (100)	2021	Drug	608	614	9.00	69.00	70.00	32.60	34.90	NA	NA	35.00	35.00
ADVOR (101)	2022	Drug	259	256	3.00	77.90	78.50	34.40	40.40	88.00	86.50	43.00	43.00
DELIVER (102)	2022	Drug	3,131	3,132	27.60	71.80	71.50	43.60	44.20	26.10	23.40	54.00	54.30
REVIVED-BCIS2 (103)	2022	Revascularization	347	353	41.00	70.00	68.80	13.00	12.00	23.00	29.00	27.00	27.00
IRONMAN (104)	2023	Drug	569	568	32.40	73.20	73.50	25.00	28.00	42.00	44.00	32.00	35.00
STRONG-HF (105)	2023	Strategy	435	425	6.00	62.90	63.00	40.00	37.00	66.00	60.00	36.70	35.90
CHAMPION-HF (106)	2023	Device	270	280	18.00	61.00	62.00	28.00	27.00	100.00	100.00	NA	NA
TRILUMINATE (107)	2023	Valvular repair	175	175	12.00	78.00	77.80	56.00	53.70	59.40	55.40	59.30	58.70
COACH (108)	2023	Strategy	2,480	2,972	9.30	78.00	78.00	45.40	45.00	NA	NA	NA	NA
MOINTOR-HF (109)	2023	Device	172	172	21.6	69	70	21.60	27.30	100.00	100.00	30.00	30.00
HEART-FID (110)	2023	Drug	1532	1533	22.8	68.6	68.6	33.00	34.60	47.80	46.40	30.80	30.60
MATTERHORN (111)	2024	Valvular repair	97	86	12.3	70.2	70.9	36.5	43.3	82.4	89.1	43.0	

© 2024 Sayed A et al. *JAMA Network Open*.

Trial (reference)	Year of publication	Type of intervention	Sample size (intervention group)	Sample size (control group)	Follow-up (months)	Age (intervention group; years)	Age (control group; years)	Females (intervention group; %)	Females (control group; %)	NYHA III/IV (intervention group; %)	NYHA III/IV (control group; %)	LVEF (intervention group; %)	LVEF (control group; %)
RESHAPE-HF2 (112)	2024	Valvular repair	250	255	18.8	70.0	69.4	22	18.2	76.4	74.1	32.0	31.0
FINEARTS-HF (113)	2024	Drug	3003	2998	32.0	71.9	72.0	45.1	45.9	30.7	31.2	52.6	52.5

NYHA: New York Heart Association Classification; LVEF: Left ventricular ejection fraction; HFrEF: Heart failure with reduced ejection fraction; HFpEF: Heart failure with preserved ejection fraction; HFmrEF: Heart failure with mildly reduced ejection fraction; NA: Not available

© 2024 Sayed A et al. *JAMA Network Open*.

eTable 2. Reasons for Excluding Articles That Qualified for Full-Text Screening

Study title	Reason for exclusion
Adherence to candesartan and placebo and outcomes in chronic heart failure in the CHARM programme: double-blind, randomised, controlled clinical trial.	A report of the CHARM trial, which has already been included.
Impact of candesartan on nonfatal myocardial infarction and cardiovascular death in patients with heart failure.	A report of the CHARM trial, which has already been included.
Effects of candesartan on mortality and morbidity in patients with chronic heart failure: the CHARM-Overall programme.	A report of the CHARM trials, which have already been included.
Effects of initiating carvedilol in patients with severe chronic heart failure: results from the COPERNICUS Study.	A report of the COPERNICUS trial, which has already been included.
Effect of metoprolol CR/XL in chronic heart failure: Metoprolol CR/XL Randomised Intervention Trial in Congestive Heart Failure (MERIT-HF).	A report of the MERIT-HF trial, which has already been included.
Heart rate as a risk factor in chronic heart failure (SHIFT): the association between heart rate and outcomes in a randomised placebo-controlled trial.	A report of the SHIFT trial, which has already been included.
Coronary-artery bypass surgery in patients with left ventricular dysfunction.	A report of the STITCHES trial, which has already been included.
Long-term use of a left ventricular assist device for end-stage heart failure.	Data only on time spent during hospitalization
Race and the response to adrenergic blockade with carvedilol in patients with chronic heart failure.	Does not report on HF hospitalizations
Efficacy of telemedical interventional management in patients with heart failure (TIM-HF2): a randomised, controlled, parallel-group, unmasked trial.	Does not report on HF hospitalizations
The angiotensin receptor neprilysin inhibitor LCZ696 in heart failure with preserved ejection fraction: a phase 2 double-blind randomised controlled trial.	Does not report on HF hospitalizations
Intrapericardial Left Ventricular Assist Device for Advanced Heart Failure.	Does not report on HF hospitalizations
Elective intra-aortic balloon counterpulsation during high-risk percutaneous coronary intervention: a randomized controlled trial.	Does not report on HF hospitalizations
Improved survival with an implanted defibrillator in patients with coronary disease at high risk for ventricular arrhythmia. Multicenter Automatic Defibrillator Implantation Trial Investigators.	Does not report on HF hospitalizations
Randomised study of effect of ibopamine on survival in patients with advanced severe heart failure. Second Prospective Randomised Study of Ibopamine on Mortality and Efficacy (PRIME II) Investigators.	Does not report on HF hospitalizations
Effect of an Emergency Department Care Bundle on 30-Day Hospital Discharge and Survival Among Elderly Patients With Acute Heart Failure: The ELISABETH Randomized Clinical Trial.	Does not report on HF hospitalizations

© 2024 Sayed A et al. *JAMA Network Open*.

Study title	Reason for exclusion
Efficacy and safety of intravenous levosimendan compared with dobutamine in severe low-output heart failure (the LIDO study): a randomised double-blind trial.	Does not report on HF hospitalizations
A comparison of oral milrinone, digoxin, and their combination in the treatment of patients with chronic heart failure.	Does not report on HF hospitalizations
Effect of short-term infusion of sodium nitroprusside on mortality rate in acute myocardial infarction complicated by left ventricular failure: results of a Veterans Administration cooperative study.	Does not report on HF hospitalizations
Evaluation study of congestive heart failure and pulmonary artery catheterization effectiveness: the ESCAPE trial.	Does not report on HF hospitalizations
Amiodarone or an implantable cardioverter-defibrillator for congestive heart failure.	Does not report on HF hospitalizations
Long-term effects of darusentan on left-ventricular remodelling and clinical outcomes in the EndothelinA Receptor Antagonist Trial in Heart Failure (EARTH): randomised, double-blind, placebo-controlled trial.	Does not report on HF hospitalizations
Effects of enalapril on mortality in severe congestive heart failure. Results of the Cooperative North Scandinavian Enalapril Survival Study (CONSENSUS).	Does not report on HF hospitalizations
Effect of ramipril on mortality and morbidity of survivors of acute myocardial infarction with clinical evidence of heart failure. The Acute Infarction Ramipril Efficacy (AIRE) Study Investigators.	Does not report on HF hospitalizations
Xamoterol in severe heart failure. The Xamoterol in Severe Heart Failure Study Group.	Does not report on HF hospitalizations
Short-term intravenous milrinone for acute exacerbation of chronic heart failure: a randomized controlled trial.	Does not report on HF hospitalizations
Levosimendan vs dobutamine for patients with acute decompensated heart failure: the SURVIVE Randomized Trial.	Does not report on HF hospitalizations
Effect of phosphodiesterase-5 inhibition on exercise capacity and clinical status in heart failure with preserved ejection fraction: a randomized clinical trial.	Does not report on HF hospitalizations
Amiodarone in patients with congestive heart failure and asymptomatic ventricular arrhythmia. Survival Trial of Antiarrhythmic Therapy in Congestive Heart Failure.	Does not report on HF hospitalizations
Comparison of carvedilol and metoprolol on clinical outcomes in patients with chronic heart failure in the Carvedilol Or Metoprolol European Trial (COMET): randomised controlled trial.	Does not report on HF hospitalizations
Ixmyelocel-T for patients with ischaemic heart failure: a prospective randomised double-blind trial.	Does not report on HF hospitalizations
Reduction of dietary sodium to less than 100 mmol in heart failure (SODIUM-HF): an international, open-label, randomised, controlled trial.	Does not report on HF hospitalizations
Intramyocardial Injection of Mesenchymal Precursor Cells and Successful Temporary Weaning From Left Ventricular Assist Device Support in Patients With Advanced Heart Failure: A Randomized Clinical Trial.	Does not report on HF hospitalizations
Continuous positive airway pressure for central sleep apnea and heart failure.	Does not report on HF hospitalizations

© 2024 Sayed A et al. *JAMA Network Open*.

Study title	Reason for exclusion
Effects of tezosentan on symptoms and clinical outcomes in patients with acute heart failure: the VERITAS randomized controlled trials.	Does not report on HF hospitalizations
EFFECTS OF UK 69 578: A NOVEL ATRIOPEPTIDASE INHIBITOR	Does not report on HF hospitalizations
BENEFIT	Does not report on HF hospitalizations
Tafamidis Treatment for Patients with Transthyretin Amyloid Cardiomyopathy	Does not report on HF hospitalizations
Effect of enalapril on myocardial infarction and unstable angina in patients with low ejection fractions.	Does not report on HF hospitalizations
Effect of carvedilol on outcome after myocardial infarction in patients with left-ventricular dysfunction: the CAPRICORN randomised trial.	Does not report on HF hospitalizations
Combined cardiac resynchronization and implantable cardioversion defibrillation in advanced chronic heart failure: the MIRACLE ICD Trial.	Does not report on HF hospitalizations separately
Beneficial effects of metoprolol in idiopathic dilated cardiomyopathy. Metoprolol in Dilated Cardiomyopathy (MDC) Trial Study Group.	Does not report on HF hospitalizations separately
Angiotensin Receptor-Neprilysin Inhibition in Acute Myocardial Infarction.	Does not report on HF hospitalizations separately
Valsartan, captopril, or both in myocardial infarction complicated by heart failure, left ventricular dysfunction, or both.	Does not report on HF hospitalizations separately
Carvedilol for children and adolescents with heart failure: a randomized controlled trial.	Does not report on HF hospitalizations separately
Serelaxin, recombinant human relaxin-2, for treatment of acute heart failure (RELAX-AHF): a randomised, placebo-controlled trial.	Does not report on HF hospitalizations separately
Relaxin for the treatment of patients with acute heart failure (Pre-RELAX-AHF): a multicentre, randomised, placebo-controlled, parallel-group, dose-finding phase IIb study.	Does not report on HF hospitalizations separately
Randomised trial of low-dose amiodarone in severe congestive heart failure. Grupo de Estudio de la Sobrevida en la Insuficiencia Cardiaca en Argentina (GESICA).	Does not report on HF hospitalizations separately
A clinical trial of the angiotensin-converting-enzyme inhibitor trandolapril in patients with left ventricular dysfunction after myocardial infarction. Trandolapril Cardiac Evaluation (TRACE) Study Group.	Does not report on HF hospitalizations separately
Efficacy and safety of exercise training in patients with chronic heart failure: HF-ACTION randomized controlled trial.	Does not report on HF hospitalizations separately
Effects of Serelaxin in Patients with Acute Heart Failure.	Does not report on HF hospitalizations separately
Results of the Medicare Health Support disease-management pilot program.	Irrelevant patient population
Irbesartan in patients with atrial fibrillation.	Irrelevant patient population
Rosiglitazone evaluated for cardiovascular outcomes--an interim analysis.	Irrelevant patient population
Effects of ramipril on cardiovascular and microvascular outcomes in people with diabetes mellitus: results of the HOPE study and	Irrelevant patient population

© 2024 Sayed A et al. *JAMA Network Open*.

Study title	Reason for exclusion
MICRO-HOPE substudy. Heart Outcomes Prevention Evaluation Study Investigators.	
Does increased access to primary care reduce hospital readmissions? Veterans Affairs Cooperative Study Group on Primary Care and Hospital Readmission.	Irrelevant patient population
Effects of oral tolvaptan in patients hospitalized for worsening heart failure: the EVEREST Outcome Trial.	No data on HF hospitalizations by group
Valsartan in a Japanese population with hypertension and other cardiovascular disease (Jikei Heart Study): a randomised, open-label, blinded endpoint morbidity-mortality study.	Retracted paper & Irrelevant patient population

© 2024 Sayed A et al. *JAMA Network Open*.

eTable 3. Bias Assessments Across Included Studies

Study	Randomization	Deviations from intended interventions	Missing outcome data	Outcome measurement	Selective Reporting	Overall
V-HeFT II	Low	Low	Low	Low	Low	Low
SOLVD-HF (B)	Low	Low	Low	Low	Low	Low
SAVE	Some Concerns	Low	Low	Low	Low	Some Concerns
SOLVD-HF (A)	Low	Low	Low	Low	Low	Low
Feldman et al., 1993	Low	Low	Low	Low	Low	Low
Rich et al., 1995	Low	Some Concerns	Low	Low	Low	Some Concerns
PRAISE-1	Low	Low	Low	Low	Low	Low
SWORD	Low	Low	Some Concerns	Low	Low	Some Concerns
ELITE	Some Concerns	Low	Low	Low	Low	Some Concerns
ANZ Trial	Low	Low	Low	Some Concerns	Low	Some Concerns
DIG trial	Low	Low	Low	Low	Low	Low
Cohn et al., 1998	Some Concerns	Low	Low	Low	Low	Some Concerns
DIAMOND-CHF	Some Concerns	Low	Low	Low	Low	Some Concerns
CIBIS-II	Low	Low	Low	Low	Low	Low
RALES	Some Concerns	Low	Low	Low	Low	Some Concerns
IMPRESS	Some Concerns	Low	Low	Low	Low	Some Concerns
DIAMOND	Low	Low	Low	Low	Low	Low
ELITE II	Some Concerns	Low	Low	Low	Low	Some Concerns
MERIT-HF	Low	Low	Low	Low	Low	Low
Val-HeFT	Low	Low	Low	Some Concerns	Low	Some Concerns
BEST	Low	Low	Low	Low	Low	Low
CAPRICORN	Low	Low	Some Concerns	Low	Low	Low
COPERNICUS	Low	Low	Low	Low	Low	Low
MUSTIC[*]	Some Concerns	Some Concerns	Low	Low	Low	Some Concerns

© 2024 Sayed A et al. *JAMA Network Open*.

Study	Randomization	Deviations from intended interventions	Missing outcome data	Outcome measurement	Selective Reporting	Overall
Troughton et al., 2000	Some Concerns	Some Concerns	Low	Some Concerns	Low	Some Concerns
MIRACLE	Low	Low	Low	Low	Low	Low
OPTIMAAL	Some Concerns	Low	Low	Low	Low	Some Concerns
VMAC	Some Concerns	Low	Low	Low	Low	Some Concerns
CHARM Preserved	Low	Low	Low	Low	Low	Low
CHARM-Alternative	Low	Low	Low	Low	Low	Low
EPHESUS	Low	Low	Low	Low	Low	Low
COMPANION	Low	Some Concerns	Low	Low	Low	Some Concerns
A-HeFT	Low	Low	Low	Low	Low	Low
ACTIV in CHF	Low	Low	Low	Low	Low	Low
CARE-HF	Low	Some Concerns	Low	Low	Low	Some Concerns
CORONA	Low	Some Concerns	Low	Low	Low	Some Concerns
GISSI-HF (Fatty Acids)	Low	Low	Low	Low	Low	Low
GISSI-HF (Rosuvastatin)	Low	Low	Low	Low	Low	Low
ANDROMEDA	Low	Low	Low	Low	Low	Low
I-PRESERVE	Low	Low	Low	Low	Low	Low
BEAUTIFUL	Low	Low	Low	Low	Low	Low
ACCLAIM	Some Concerns	Low	Low	Low	Low	Some Concerns
AF-CHF	Low	Low	Low	Low	Low	Low
TIME-CHF	Low	Some Concerns	Some Concerns	Some Concerns	Low	Some Concerns
MADIT-CRT	Low	Low	Low	Low	Low	Low
STITCH 2	Low	Low	Low	Some Concerns	Low	Some Concerns
HEAAL	Some Concerns	Low	Low	Low	Low	Some Concerns
FAIR-HF	Low	Low	Some Concerns	Low	Low	Some Concerns
RAFT	Low	Low	Low	Low	Low	Low
SHIFT	Low	Low	Low	Low	Low	Low

© 2024 Sayed A et al. *JAMA Network Open*.

Study	Randomization	Deviations from intended interventions	Missing outcome data	Outcome measurement	Selective Reporting	Overall
PROTECT	Low	Low	Low	Low	Low	Low
HART	Low	Some Concerns	Low	Low	Low	Some Concerns
Tele-HF	Low	Some Concerns	Low	Low	Low	Some Concerns
ASCNED-HF	Low	Some Concerns	Low	Low	Low	Some Concerns
EMPHASIS HF	Low	Low	Low	Low	Low	Low
TIME	Some Concerns	High	Low	Low	Low	High
FOCUS-CCTRN	Some Concerns	Low	Low	Low	Low	Some Concerns
HF-ACTION	Low	Some Concerns	Low	Low	Low	Some Concerns
CARRESS	Low	Low	Low	Low	Low	Low
WARCEF	Low	Low	Low	Low	Low	Low
BLOCK HF	Low	Low	Low	Low	Low	Low
EchoCRT	Low	Low	Low	Low	Low	Low
ASTRONAUT	Some Concerns	Low	Low	Low	Low	Some Concerns
CELLWAVE	Some Concerns	Low	Low	Low	Low	Some Concerns
CHARM-Added	Low	Low	Low	Low	Low	Low
RED-HF	Low	Low	Low	Low	Low	Low
PARADIGM-HF	Low	Low	Low	Low	Low	Low
IN-TIME	Low	Some Concerns	Low	Low	Low	Some Concerns
TOPCAT	Low	Low	Low	Low	Low	Low
TAC-HFT	Some Concerns	Some Concerns	Low	Low	Low	Some Concerns
SERVE-HF	Low	Some Concerns	Low	Low	Low	Some Concerns
SOCRATES	Low	Low	Low	Low	Low	Low
ATMOSPHERE	Low	Low	Low	Low	Low	Low
CUPID 2	Low	Low	Low	Low	Low	Low
COSMIC-HF	Low	Low	Low	Some Concerns	Low	Some Concerns
STICHES	Low	Low	Low	Some Concerns	Low	Some Concerns

© 2024 Sayed A et al. *JAMA Network Open*.

Study	Randomization	Deviations from intended interventions	Missing outcome data	Outcome measurement	Selective Reporting	Overall
MOOD-HF	Low	Low	Low	Low	Low	Low
FIGHT	Low	Low	Some Concerns	Low	Low	Low
GUIDE-IT	Low	Some Concerns	Low	Low	Low	Some Concerns
TRUE-AHF	Low	Low	Low	Low	Low	Low
CASTLE-AF	Low	Some Concerns	Some Concerns	Low	Low	Some Concerns
CULPRIT-SHOCK	Low	Some Concerns	Low	Low	Low	Some Concerns
MITRA FR	Low	Some Concerns	Low	Low	Low	Some Concerns
COMMANDER HF	Low	Low	Low	Low	Low	Low
COAPT	Low	Some Concerns	Low	Low	Low	Some Concerns
PIONEER-HF	Some Concerns	Low	Low	Low	Low	Some Concerns
PARAGON-HF	Low	Low	Low	Low	Low	Low
DAPA-HF	Low	Low	Low	Low	Low	Low
GALACTIC	Low	Some Concerns	Low	Low	Low	Some Concerns
PANACHE	Low	Low	Low	Low	Low	Low
PACT-HF[†]	Low	Some Concerns	Low	Low	Low	Some Concerns
EMPEROR-REDUCED	Low	Low	Low	Low	Low	Low
AFFIRM-AHF	Low	Some Concerns	Low	Low	Low	Some Concerns
VICTORIA	Low	Low	Low	Low	Low	Low
GALACTIC-HF	Low	Low	Low	Low	Low	Low
CONNECT-HF	Low	Some Concerns	Low	Low	Low	Some Concerns
EMPEROR-Preserved	Low	Low	Low	Low	Low	Low
GUIDE-HF	Some Concerns	Low	Low	Low	Low	Some Concerns
REHAB-HF	Low	Some Concerns	Low	Low	Low	Some Concerns
SOLOIST-WHF	Low	Low	Low	Low	Low	Low
ADVOR	Low	Low	Low	Low	Low	Low
DELIVER	Low	Low	Low	Low	Low	Low

© 2024 Sayed A et al. *JAMA Network Open*.

Study	Randomization	Deviations from intended interventions	Missing outcome data	Outcome measurement	Selective Reporting	Overall
REVIVED-BCIS2	Low	Some Concerns	Low	Low	Low	Some Concerns
IRONMAN	Low	Low	Low	Low	Low	Low
STRONG-HF	Low	Low	Low	Low	Low	Low
CHAMPION-HF	Low	Low	Low	Low	Low	Low
TRILUMINATE	Some Concerns	Low	Low	Low	Low	Some Concerns
COACH[†]	Low	Low	Low	Low	Low	Some Concerns
MONITOR-HF	Low	Some Concerns	Low	Some Concerns	Low	Some Concerns
HEART-FID	Low	Low	Low	Low	Low	Low
MATTERHORN	Low	Low	Low	Low	Low	Low
RESHAPE-HF2	Low	Low	Low	Low	Low	Low
FINEARTS-HF	Low	Low	Low	Low	Low	Low

[*]Because this was a cross-over trial, an additional bias domain, bias arising due to period or carryover effects, was considered and rated as having a low risk of bias.

[†]Because these were cluster trials, an additional bias domain, bias arising due to recruitment of patients into clusters, was considered and rated as having some concerns of bias.

© 2024 Sayed A et al. *JAMA Network Open*.

eFigure 1. PRISMA Diagram Outlining the Study Selection Process

All studies were eligible for the reporting of all-cause hospitalization analysis. Studies were eligible for the ratio of HF to all-cause hospitalization and predictiveness of HF for all-cause hospitalization analyses if they reported on the number of patients who experienced HF and all-cause hospitalizations.

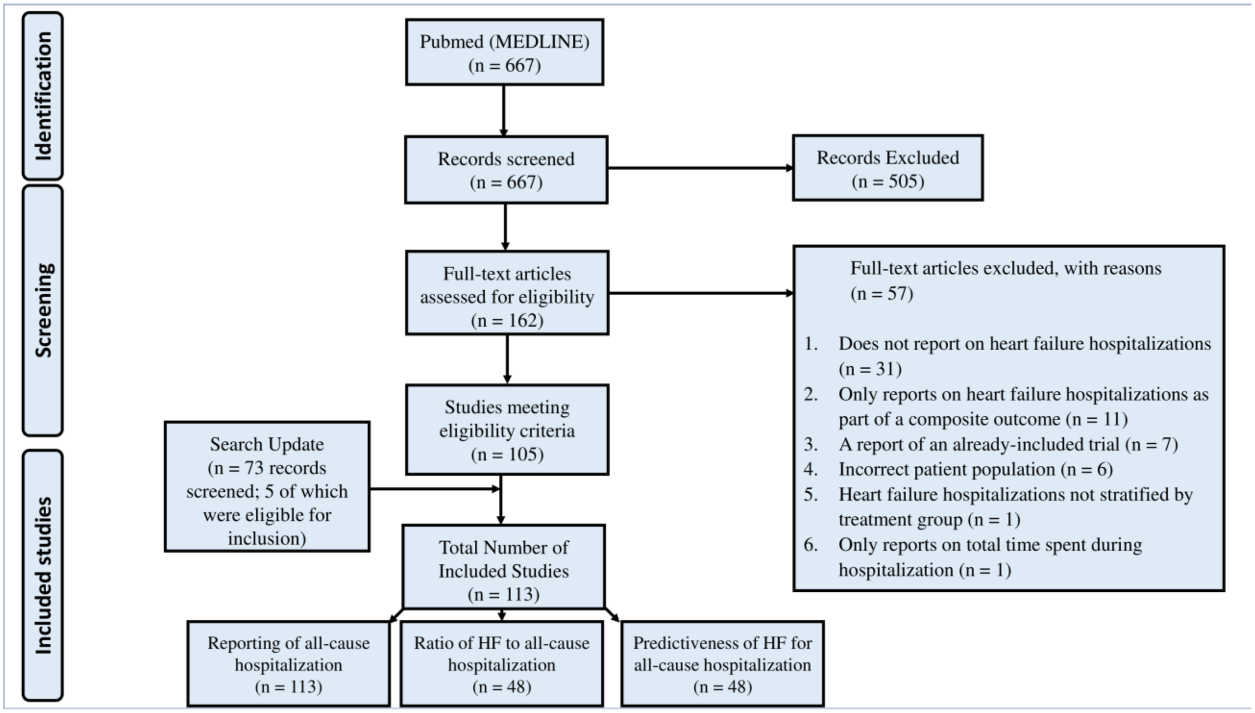

© 2024 Sayed A et al. *JAMA Network Open*.

eFigure 2. Correlation Between Treatment Effects on Heart Failure and All-Cause Hospitalization Using Risk Ratios

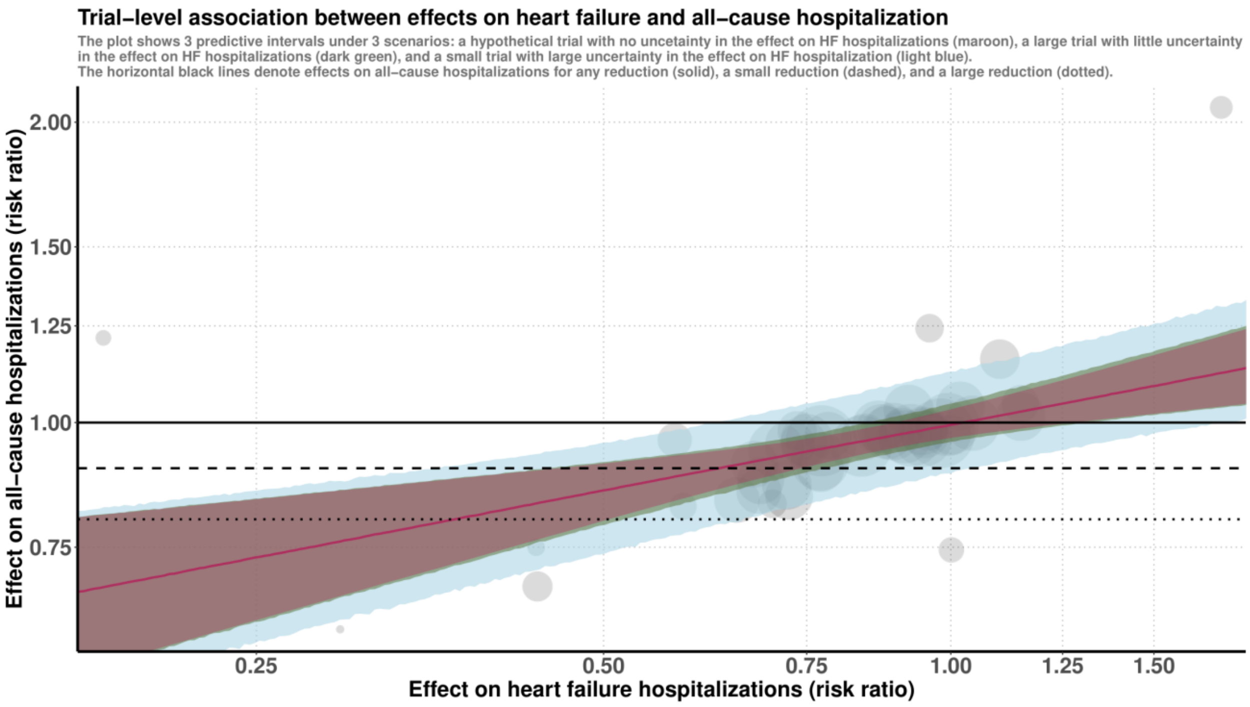

© 2024 Sayed A et al. *JAMA Network Open*.

eFigure 3. Correlation Between Treatment Effects on Heart Failure and All-Cause Hospitalization Using Absolute Risk Differences

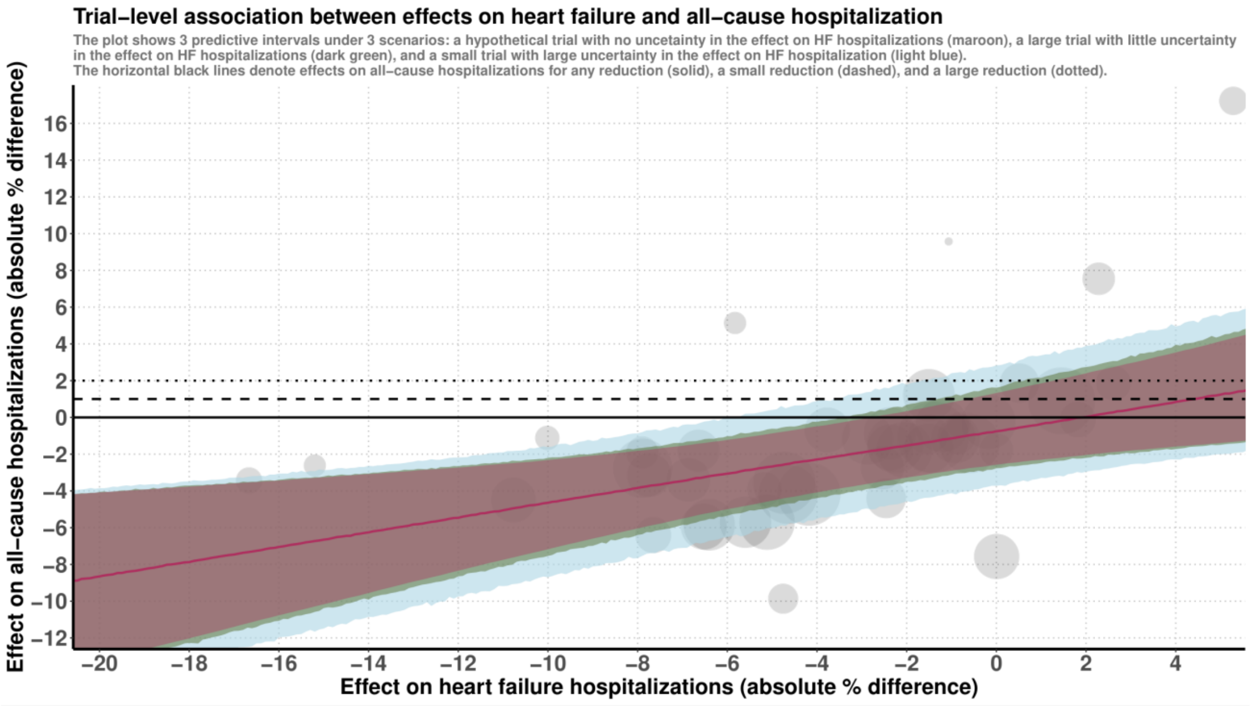

© 2024 Sayed A et al. *JAMA Network Open*.

eReferences in eMethods

1. Cohn JN, Johnson G, Ziesche S, et al. A comparison of enalapril with hydralazine-isosorbide dinitrate in the treatment of chronic congestive heart failure. N Engl J Med. Aug 1 1991;325(5):303-10. doi:10.1056/nejm199108013250502

2. Yusuf S, Pitt B, Davis CE, Hood WB, Cohn JN. Effect of enalapril on survival in patients with reduced left ventricular ejection fractions and congestive heart failure. N Engl J Med. Aug 1 1991;325(5):293-302. doi:10.1056/nejm199108013250501

3. Pfeffer MA, Braunwald E, Moye LA, et al. Effect of captopril on mortality and morbidity in patients with left ventricular dysfunction after myocardial infarction. Results of the survival and ventricular enlargement trial. The SAVE Investigators. N Engl J Med. Sep 3 1992;327(10):669-77. doi:10.1056/nejm199209033271001

4. Yusuf S, Pitt B, Davis CE, Hood WB, Jr., Cohn JN. Effect of enalapril on mortality and the development of heart failure in asymptomatic patients with reduced left ventricular ejection fractions. N Engl J Med. Sep 3 1992;327(10):685-91. doi:10.1056/nejm199209033271003

5. Feldman AM, Bristow MR, Parmley WW, et al. Effects of vesnarinone on morbidity and mortality in patients with heart failure. Vesnarinone Study Group. N Engl J Med. Jul 15 1993;329(3):149-55. doi:10.1056/nejm199307153290301

6. Rich MW, Beckham V, Wittenberg C, Leven CL, Freedland KE, Carney RM. A multidisciplinary intervention to prevent the readmission of elderly patients with congestive heart failure. N Engl J Med. Nov 2 1995;333(18):1190-5. doi:10.1056/nejm199511023331806

© 2024 Sayed A et al. *JAMA Network Open*.

7. Packer M, O'Connor CM, Ghali JK, et al. Effect of amlodipine on morbidity and mortality in severe chronic heart failure. Prospective Randomized Amlodipine Survival Evaluation Study Group. N Engl J Med. Oct 10 1996;335(15):1107-14. doi:10.1056/nejm199610103351504

8. Waldo AL, Camm AJ, deRuyter H, et al. Effect of d-sotalol on mortality in patients with left ventricular dysfunction after recent and remote myocardial infarction. The SWORD Investigators. Survival With Oral d-Sotalol. Lancet. Jul 6 1996;348(9019):7-12. doi:10.1016/s0140-6736(96)02149-6

9. Pitt B, Segal R, Martinez FA, et al. Randomised trial of losartan versus captopril in patients over 65 with heart failure (Evaluation of Losartan in the Elderly Study, ELITE). Lancet. Mar 15 1997;349(9054):747-52. doi:10.1016/s0140-6736(97)01187-2

10. Randomised, placebo-controlled trial of carvedilol in patients with congestive heart failure due to ischaemic heart disease. Australia/New Zealand Heart Failure Research Collaborative Group. Lancet. Feb 8 1997;349(9049):375-80.

11. The effect of digoxin on mortality and morbidity in patients with heart failure. N Engl J Med. Feb 20 1997;336(8):525-33. doi:10.1056/nejm199702203360801

12. Cohn JN, Goldstein SO, Greenberg BH, et al. A dose-dependent increase in mortality with vesnarinone among patients with severe heart failure. Vesnarinone Trial Investigators. N Engl J Med. Dec 17 1998;339(25):1810-6. doi:10.1056/nejm199812173392503

13. Torp-Pedersen C, Moller M, Bloch-Thomsen PE, et al. Dofetilide in patients with congestive heart failure and left ventricular dysfunction. Danish Investigations of Arrhythmia and Mortality on Dofetilide Study Group. N Engl J Med. Sep 16 1999;341(12):857-65. doi:10.1056/nejm199909163411201

© 2024 Sayed A et al. *JAMA Network Open*.

14. The Cardiac Insufficiency Bisoprolol Study II (CIBIS-II): a randomised trial. Lancet. Jan 2 1999;353(9146):9-13.

15. Pitt B, Zannad F, Remme WJ, et al. The effect of spironolactone on morbidity and mortality in patients with severe heart failure. Randomized Aldactone Evaluation Study Investigators. N Engl J Med. Sep 2 1999;341(10):709-17. doi:10.1056/nejm199909023411001

16. Rouleau JL, Pfeffer MA, Stewart DJ, et al. Comparison of vasopeptidase inhibitor, omapatrilat, and lisinopril on exercise tolerance and morbidity in patients with heart failure: IMPRESS randomised trial. Lancet. Aug 19 2000;356(9230):615-20. doi:10.1016/s0140-6736(00)02602-7

17. Kober L, Bloch Thomsen PE, Moller M, et al. Effect of dofetilide in patients with recent myocardial infarction and left-ventricular dysfunction: a randomised trial. Lancet. Dec 16 2000;356(9247):2052-8. doi:10.1016/s0140-6736(00)03402-4

18. Pitt B, Poole-Wilson PA, Segal R, et al. Effect of losartan compared with captopril on mortality in patients with symptomatic heart failure: randomised trial--the Losartan Heart Failure Survival Study ELITE II. Lancet. May 6 2000;355(9215):1582-7. doi:10.1016/s0140-6736(00)02213-3

19. Hjalmarson A, Goldstein S, Fagerberg B, et al. Effects of controlled-release metoprolol on total mortality, hospitalizations, and well-being in patients with heart failure: the Metoprolol CR/XL Randomized Intervention Trial in congestive heart failure (MERIT-HF). MERIT-HF Study Group. Jama. Mar 8 2000;283(10):1295-302. doi:10.1001/jama.283.10.1295

20. Troughton RW, Frampton CM, Yandle TG, Espiner EA, Nicholls MG, Richards AM. Treatment of heart failure guided by plasma aminoterminal brain natriuretic peptide (N-BNP) concentrations. Lancet. Apr 1 2000;355(9210):1126-30. doi:10.1016/s0140-6736(00)02060-2

© 2024 Sayed A et al. *JAMA Network Open*.

21. Cohn JN, Tognoni G. A randomized trial of the angiotensin-receptor blocker valsartan in chronic heart failure. N Engl J Med. Dec 6 2001;345(23):1667-75. doi:10.1056/NEJMoa010713

22. Eichhorn EJ, Domanski MJ, Krause-Steinrauf H, Bristow MR, Lavori PW. A trial of the beta-blocker bucindolol in patients with advanced chronic heart failure. N Engl J Med. May 31 2001;344(22):1659-67. doi:10.1056/nejm200105313442202

23. Dargie HJ. Effect of carvedilol on outcome after myocardial infarction in patients with left-ventricular dysfunction: the CAPRICORN randomised trial. Lancet. May 5 2001;357(9266):1385-90. doi:10.1016/s0140-6736(00)04560-8

24. Packer M, Coats AJ, Fowler MB, et al. Effect of carvedilol on survival in severe chronic heart failure. N Engl J Med. May 31 2001;344(22):1651-8. doi:10.1056/nejm200105313442201

25. Cazeau S, Leclercq C, Lavergne T, et al. Effects of multisite biventricular pacing in patients with heart failure and intraventricular conduction delay. N Engl J Med. Mar 22 2001;344(12):873-80. doi:10.1056/nejm200103223441202

26. Abraham WT, Fisher WG, Smith AL, et al. Cardiac resynchronization in chronic heart failure. N Engl J Med. Jun 13 2002;346(24):1845-53. doi:10.1056/NEJMoa013168

27. Dickstein K, Kjekshus J. Effects of losartan and captopril on mortality and morbidity in high-risk patients after acute myocardial infarction: the OPTIMAAL randomised trial. Optimal Trial in Myocardial Infarction with Angiotensin II Antagonist Losartan. Lancet. Sep 7 2002;360(9335):752-60. doi:10.1016/s0140-6736(02)09895-1

28. Intravenous nesiritide vs nitroglycerin for treatment of decompensated congestive heart failure: a randomized controlled trial. Jama. Mar 27 2002;287(12):1531-40. doi:10.1001/jama.287.12.1531

© 2024 Sayed A et al. *JAMA Network Open*.

29. Yusuf S, Pfeffer MA, Swedberg K, et al. Effects of candesartan in patients with chronic heart failure and preserved left-ventricular ejection fraction: the CHARM-Preserved Trial. Lancet. Sep 6 2003;362(9386):777-81. doi:10.1016/s0140-6736(03)14285-7

30. Granger CB, McMurray JJ, Yusuf S, et al. Effects of candesartan in patients with chronic heart failure and reduced left-ventricular systolic function intolerant to angiotensin-converting-enzyme inhibitors: the CHARM-Alternative trial. Lancet. Sep 6 2003;362(9386):772-6. doi:10.1016/s0140-6736(03)14284-5

31. McMurray JJ, Ostergren J, Swedberg K, et al. Effects of candesartan in patients with chronic heart failure and reduced left-ventricular systolic function taking angiotensin-converting-enzyme inhibitors: the CHARM-Added trial. Lancet. Sep 6 2003;362(9386):767-71. doi:10.1016/s0140-6736(03)14283-3

32. Pitt B, Remme W, Zannad F, et al. Eplerenone, a selective aldosterone blocker, in patients with left ventricular dysfunction after myocardial infarction. N Engl J Med. Apr 3 2003;348(14):1309-21. doi:10.1056/NEJMoa030207

33. Bristow MR, Saxon LA, Boehmer J, et al. Cardiac-resynchronization therapy with or without an implantable defibrillator in advanced chronic heart failure. N Engl J Med. May 20 2004;350(21):2140-50. doi:10.1056/NEJMoa032423

34. Taylor AL, Ziesche S, Yancy C, et al. Combination of isosorbide dinitrate and hydralazine in blacks with heart failure. N Engl J Med. Nov 11 2004;351(20):2049-57. doi:10.1056/NEJMoa042934

35. Gheorghiade M, Gattis WA, O'Connor CM, et al. Effects of tolvaptan, a vasopressin antagonist, in patients hospitalized with worsening heart failure: a randomized controlled trial. Jama. Apr 28 2004;291(16):1963-71. doi:10.1001/jama.291.16.1963

© 2024 Sayed A et al. *JAMA Network Open*.

36. Cleland JG, Daubert JC, Erdmann E, et al. The effect of cardiac resynchronization on morbidity and mortality in heart failure. N Engl J Med. Apr 14 2005;352(15):1539-49. doi:10.1056/NEJMoa050496

37. Kjekshus J, Apetrei E, Barrios V, et al. Rosuvastatin in older patients with systolic heart failure. N Engl J Med. Nov 29 2007;357(22):2248-61. doi:10.1056/NEJMoa0706201

38. Tavazzi L, Maggioni AP, Marchioli R, et al. Effect of n-3 polyunsaturated fatty acids in patients with chronic heart failure (the GISSI-HF trial): a randomised, double-blind, placebo-controlled trial. Lancet. Oct 4 2008;372(9645):1223-30. doi:10.1016/s0140-6736(08)61239-8

39. Tavazzi L, Maggioni AP, Marchioli R, et al. Effect of rosuvastatin in patients with chronic heart failure (the GISSI-HF trial): a randomised, double-blind, placebo-controlled trial. Lancet. Oct 4 2008;372(9645):1231-9. doi:10.1016/s0140-6736(08)61240-4

40. Kober L, Torp-Pedersen C, McMurray JJ, et al. Increased mortality after dronedarone therapy for severe heart failure. N Engl J Med. Jun 19 2008;358(25):2678-87. doi:10.1056/NEJMoa0800456

41. Massie BM, Carson PE, McMurray JJ, et al. Irbesartan in patients with heart failure and preserved ejection fraction. N Engl J Med. Dec 4 2008;359(23):2456-67. doi:10.1056/NEJMoa0805450

42. Fox K, Ford I, Steg PG, Tendera M, Ferrari R. Ivabradine for patients with stable coronary artery disease and left-ventricular systolic dysfunction (BEAUTIFUL): a randomised, double-blind, placebo-controlled trial. Lancet. Sep 6 2008;372(9641):807-16. doi:10.1016/s0140-6736(08)61170-8

43. Torre-Amione G, Anker SD, Bourge RC, et al. Results of a non-specific immunomodulation therapy in chronic heart failure (ACCLAIM trial): a placebo-controlled randomised trial. Lancet. Jan 19 2008;371(9608):228-36. doi:10.1016/s0140-6736(08)60134-8

© 2024 Sayed A et al. *JAMA Network Open*.

44. Roy D, Talajic M, Nattel S, et al. Rhythm Control versus Rate Control for Atrial Fibrillation and Heart Failure. New England Journal of Medicine. 2008/06/19 2008;358(25):2667-2677. doi:10.1056/NEJMoa0708789

45. Pfisterer M, Buser P, Rickli H, et al. BNP-guided vs symptom-guided heart failure therapy: the Trial of Intensified vs Standard Medical Therapy in Elderly Patients With Congestive Heart Failure (TIME-CHF) randomized trial. Jama. Jan 28 2009;301(4):383-92. doi:10.1001/jama.2009.2

46. Moss AJ, Hall WJ, Cannom DS, et al. Cardiac-resynchronization therapy for the prevention of heart-failure events. N Engl J Med. Oct 1 2009;361(14):1329-38. doi:10.1056/NEJMoa0906431

47. Jones RH, Velazquez EJ, Michler RE, et al. Coronary bypass surgery with or without surgical ventricular reconstruction. N Engl J Med. Apr 23 2009;360(17):1705-17. doi:10.1056/NEJMoa0900559

48. Konstam MA, Neaton JD, Dickstein K, et al. Effects of high-dose versus low-dose losartan on clinical outcomes in patients with heart failure (HEAAL study): a randomised, double-blind trial. Lancet. Nov 28 2009;374(9704):1840-8. doi:10.1016/s0140-6736(09)61913-9

49. Anker SD, Comin Colet J, Filippatos G, et al. Ferric carboxymaltose in patients with heart failure and iron deficiency. N Engl J Med. Dec 17 2009;361(25):2436-48. doi:10.1056/NEJMoa0908355

50. Tang AS, Wells GA, Talajic M, et al. Cardiac-resynchronization therapy for mild-to-moderate heart failure. N Engl J Med. Dec 16 2010;363(25):2385-95. doi:10.1056/NEJMoa1009540

51. Swedberg K, Komajda M, Bohm M, et al. Ivabradine and outcomes in chronic heart failure (SHIFT): a randomised placebo-controlled study. Lancet. Sep 11 2010;376(9744):875-85. doi:10.1016/s0140-6736(10)61198-1

52. Massie BM, O'Connor CM, Metra M, et al. Rolofylline, an adenosine A1-receptor antagonist, in acute heart failure. N Engl J Med. Oct 7 2010;363(15):1419-28. doi:10.1056/NEJMoa0912613

53. Powell LH, Calvin JE, Jr., Richardson D, et al. Self-management counseling in patients with heart failure: the heart failure adherence and retention randomized behavioral trial. Jama. Sep 22 2010;304(12):1331-8. doi:10.1001/jama.2010.1362

54. Chaudhry SI, Mattera JA, Curtis JP, et al. Telemonitoring in patients with heart failure. N Engl J Med. Dec 9 2010;363(24):2301-9. doi:10.1056/NEJMoa1010029

55. O'Connor CM, Starling RC, Hernandez AF, et al. Effect of nesiritide in patients with acute decompensated heart failure. N Engl J Med. Jul 7 2011;365(1):32-43. doi:10.1056/NEJMoa1100171

56. Zannad F, McMurray JJ, Krum H, et al. Eplerenone in patients with systolic heart failure and mild symptoms. N Engl J Med. Jan 6 2011;364(1):11-21. doi:10.1056/NEJMoa1009492

57. Abraham WT, Adamson PB, Bourge RC, et al. Wireless pulmonary artery haemodynamic monitoring in chronic heart failure: a randomised controlled trial. Lancet. Feb 19 2011;377(9766):658-66. doi:10.1016/S0140-6736(11)60101-3

58. Traverse JH, Henry TD, Pepine CJ, et al. Effect of the use and timing of bone marrow mononuclear cell delivery on left ventricular function after acute myocardial infarction: the TIME randomized trial. Jama. Dec 12 2012;308(22):2380-9. doi:10.1001/jama.2012.28726

59. Perin EC, Willerson JT, Pepine CJ, et al. Effect of transendocardial delivery of autologous bone marrow mononuclear cells on functional capacity, left ventricular function, and perfusion in chronic heart failure: the FOCUS-CCTRN trial. Jama. Apr 25 2012;307(16):1717-26. doi:10.1001/jama.2012.418

60. Blumenthal JA, Babyak MA, O'Connor C, et al. Effects of exercise training on depressive symptoms in patients with chronic heart failure: the HF-ACTION randomized trial. Jama. Aug 1 2012;308(5):465-74. doi:10.1001/jama.2012.8720

© 2024 Sayed A et al. *JAMA Network Open*.

61. Bart BA, Goldsmith SR, Lee KL, et al. Ultrafiltration in decompensated heart failure with cardiorenal syndrome. N Engl J Med. Dec 13 2012;367(24):2296-304. doi:10.1056/NEJMoa1210357

62. Homma S, Thompson JL, Pullicino PM, et al. Warfarin and aspirin in patients with heart failure and sinus rhythm. N Engl J Med. May 17 2012;366(20):1859-69. doi:10.1056/NEJMoa1202299

63. Curtis AB, Worley SJ, Adamson PB, et al. Biventricular pacing for atrioventricular block and systolic dysfunction. N Engl J Med. Apr 25 2013;368(17):1585-93. doi:10.1056/NEJMoa1210356

64. Ruschitzka F, Abraham WT, Singh JP, et al. Cardiac-resynchronization therapy in heart failure with a narrow QRS complex. N Engl J Med. Oct 10 2013;369(15):1395-405. doi:10.1056/NEJMoa1306687

65. Gheorghiade M, Bohm M, Greene SJ, et al. Effect of aliskiren on postdischarge mortality and heart failure readmissions among patients hospitalized for heart failure: the ASTRONAUT randomized trial. Jama. Mar 20 2013;309(11):1125-35. doi:10.1001/jama.2013.1954

66. Assmus B, Walter DH, Seeger FH, et al. Effect of shock wave-facilitated intracoronary cell therapy on LVEF in patients with chronic heart failure: the CELLWAVE randomized clinical trial. Jama. Apr 17 2013;309(15):1622-31. doi:10.1001/jama.2013.3527

67. Swedberg K, Young JB, Anand IS, et al. Treatment of anemia with darbepoetin alfa in systolic heart failure. N Engl J Med. Mar 28 2013;368(13):1210-9. doi:10.1056/NEJMoa1214865

68. McMurray JJ, Packer M, Desai AS, et al. Angiotensin-neprilysin inhibition versus enalapril in heart failure. N Engl J Med. Sep 11 2014;371(11):993-1004. doi:10.1056/NEJMoa1409077

69. Hindricks G, Taborsky M, Glikson M, et al. Implant-based multiparameter telemonitoring of patients with heart failure (IN-TIME): a randomised controlled trial. Lancet. Aug 16 2014;384(9943):583-590. doi:10.1016/s0140-6736(14)61176-4

© 2024 Sayed A et al. *JAMA Network Open*.

70. Pitt B, Pfeffer MA, Assmann SF, et al. Spironolactone for heart failure with preserved ejection fraction. N Engl J Med. Apr 10 2014;370(15):1383-92. doi:10.1056/NEJMoa1313731

71. Heldman AW, DiFede DL, Fishman JE, et al. Transendocardial mesenchymal stem cells and mononuclear bone marrow cells for ischemic cardiomyopathy: the TAC-HFT randomized trial. Jama. Jan 1 2014;311(1):62-73. doi:10.1001/jama.2013.282909

72. Cowie MR, Woehrle H, Wegscheider K, et al. Adaptive Servo-Ventilation for Central Sleep Apnea in Systolic Heart Failure. N Engl J Med. Sep 17 2015;373(12):1095-105. doi:10.1056/NEJMoa1506459

73. Gheorghiade M, Greene SJ, Butler J, et al. Effect of Vericiguat, a Soluble Guanylate Cyclase Stimulator, on Natriuretic Peptide Levels in Patients With Worsening Chronic Heart Failure and Reduced Ejection Fraction: The SOCRATES-REDUCED Randomized Trial. Jama. Dec 1 2015;314(21):2251-62. doi:10.1001/jama.2015.15734

74. McMurray JJ, Krum H, Abraham WT, et al. Aliskiren, Enalapril, or Aliskiren and Enalapril in Heart Failure. N Engl J Med. Apr 21 2016;374(16):1521-32. doi:10.1056/NEJMoa1514859

75. Greenberg B, Butler J, Felker GM, et al. Calcium upregulation by percutaneous administration of gene therapy in patients with cardiac disease (CUPID 2): a randomised, multinational, double-blind, placebo-controlled, phase 2b trial. Lancet. Mar 19 2016;387(10024):1178-86. doi:10.1016/S0140-6736(16)00082-9

76. Teerlink JR, Felker GM, McMurray JJ, et al. Chronic Oral Study of Myosin Activation to Increase Contractility in Heart Failure (COSMIC-HF): a phase 2, pharmacokinetic, randomised, placebo-controlled trial. Lancet. Dec 10 2016;388(10062):2895-2903. doi:10.1016/s0140-6736(16)32049-9

© 2024 Sayed A et al. *JAMA Network Open*.

77. Velazquez EJ, Lee KL, Jones RH, et al. Coronary-Artery Bypass Surgery in Patients with Ischemic Cardiomyopathy. N Engl J Med. Apr 21 2016;374(16):1511-20. doi:10.1056/NEJMoa1602001

78. Angermann CE, Gelbrich G, Stork S, et al. Effect of Escitalopram on All-Cause Mortality and Hospitalization in Patients With Heart Failure and Depression: The MOOD-HF Randomized Clinical Trial. Jama. Jun 28 2016;315(24):2683-93. doi:10.1001/jama.2016.7635

79. Margulies KB, Hernandez AF, Redfield MM, et al. Effects of Liraglutide on Clinical Stability Among Patients With Advanced Heart Failure and Reduced Ejection Fraction: A Randomized Clinical Trial. Jama. Aug 2 2016;316(5):500-8. doi:10.1001/jama.2016.10260

80. Felker GM, Anstrom KJ, Adams KF, et al. Effect of Natriuretic Peptide-Guided Therapy on Hospitalization or Cardiovascular Mortality in High-Risk Patients With Heart Failure and Reduced Ejection Fraction: A Randomized Clinical Trial. Jama. Aug 22 2017;318(8):713-720. doi:10.1001/jama.2017.10565

81. Packer M, O'Connor C, McMurray JJV, et al. Effect of Ularitide on Cardiovascular Mortality in Acute Heart Failure. N Engl J Med. May 18 2017;376(20):1956-1964. doi:10.1056/NEJMoa1601895

82. Marrouche NF, Brachmann J, Andresen D, et al. Catheter Ablation for Atrial Fibrillation with Heart Failure. N Engl J Med. Feb 1 2018;378(5):417-427. doi:10.1056/NEJMoa1707855

83. Thiele H, Akin I, Sandri M, et al. One-Year Outcomes after PCI Strategies in Cardiogenic Shock. N Engl J Med. Nov 1 2018;379(18):1699-1710. doi:10.1056/NEJMoa1808788

84. Obadia JF, Messika-Zeitoun D, Leurent G, et al. Percutaneous Repair or Medical Treatment for Secondary Mitral Regurgitation. N Engl J Med. Dec 13 2018;379(24):2297-2306. doi:10.1056/NEJMoa1805374

© 2024 Sayed A et al. *JAMA Network Open*.

85. Zannad F, Anker SD, Byra WM, et al. Rivaroxaban in Patients with Heart Failure, Sinus Rhythm, and Coronary Disease. N Engl J Med. Oct 4 2018;379(14):1332-1342. doi:10.1056/NEJMoa1808848

86. Stone GW, Lindenfeld J, Abraham WT, et al. Transcatheter Mitral-Valve Repair in Patients with Heart Failure. N Engl J Med. Dec 13 2018;379(24):2307-2318. doi:10.1056/NEJMoa1806640

87. Velazquez EJ, Morrow DA, DeVore AD, et al. Angiotensin-Neprilysin Inhibition in Acute Decompensated Heart Failure. N Engl J Med. Feb 7 2019;380(6):539-548. doi:10.1056/NEJMoa1812851

88. Solomon SD, McMurray JJV, Anand IS, et al. Angiotensin-Neprilysin Inhibition in Heart Failure with Preserved Ejection Fraction. N Engl J Med. Oct 24 2019;381(17):1609-1620. doi:10.1056/NEJMoa1908655

89. McMurray JJV, Solomon SD, Inzucchi SE, et al. Dapagliflozin in Patients with Heart Failure and Reduced Ejection Fraction. N Engl J Med. Nov 21 2019;381(21):1995-2008. doi:10.1056/NEJMoa1911303

90. Kozhuharov N, Goudev A, Flores D, et al. Effect of a Strategy of Comprehensive Vasodilation vs Usual Care on Mortality and Heart Failure Rehospitalization Among Patients With Acute Heart Failure: The GALACTIC Randomized Clinical Trial. Jama. Dec 17 2019;322(23):2292-2302. doi:10.1001/jama.2019.18598

91. Shah SJ, Voors AA, McMurray JJV, et al. Effect of Neladenoson Bialanate on Exercise Capacity Among Patients With Heart Failure With Preserved Ejection Fraction: A Randomized Clinical Trial. Jama. Jun 4 2019;321(21):2101-2112. doi:10.1001/jama.2019.6717

92. Van Spall HGC, Lee SF, Xie F, et al. Effect of Patient-Centered Transitional Care Services on Clinical Outcomes in Patients Hospitalized for Heart Failure: The PACT-HF Randomized Clinical Trial. Jama. Feb 26 2019;321(8):753-761. doi:10.1001/jama.2019.0710

© 2024 Sayed A et al. *JAMA Network Open*.

93. Packer M, Anker SD, Butler J, et al. Cardiovascular and Renal Outcomes with Empagliflozin in Heart Failure. N Engl J Med. Oct 8 2020;383(15):1413-1424. doi:10.1056/NEJMoa2022190

94. Ponikowski P, Kirwan BA, Anker SD, et al. Ferric carboxymaltose for iron deficiency at discharge after acute heart failure: a multicentre, double-blind, randomised, controlled trial. Lancet. Dec 12 2020;396(10266):1895-1904. doi:10.1016/s0140-6736(20)32339-4

95. Armstrong PW, Pieske B, Anstrom KJ, et al. Vericiguat in Patients with Heart Failure and Reduced Ejection Fraction. N Engl J Med. May 14 2020;382(20):1883-1893. doi:10.1056/NEJMoa1915928

96. Teerlink JR, Diaz R, Felker GM, et al. Cardiac Myosin Activation with Omecamtiv Mecarbil in Systolic Heart Failure. N Engl J Med. Jan 14 2021;384(2):105-116. doi:10.1056/NEJMoa2025797

97. DeVore AD, Granger BB, Fonarow GC, et al. Effect of a Hospital and Postdischarge Quality Improvement Intervention on Clinical Outcomes and Quality of Care for Patients With Heart Failure With Reduced Ejection Fraction: The CONNECT-HF Randomized Clinical Trial. Jama. Jul 27 2021;326(4):314-323. doi:10.1001/jama.2021.8844

98. Anker SD, Butler J, Filippatos G, et al. Empagliflozin in Heart Failure with a Preserved Ejection Fraction. N Engl J Med. Oct 14 2021;385(16):1451-1461. doi:10.1056/NEJMoa2107038

99. Lindenfeld J, Zile MR, Desai AS, et al. Haemodynamic-guided management of heart failure (GUIDE-HF): a randomised controlled trial. Lancet. Sep 11 2021;398(10304):991-1001. doi:10.1016/s0140-6736(21)01754-2

100. Kitzman DW, Whellan DJ, Duncan P, et al. Physical Rehabilitation for Older Patients Hospitalized for Heart Failure. N Engl J Med. Jul 15 2021;385(3):203-216. doi:10.1056/NEJMoa2026141

© 2024 Sayed A et al. *JAMA Network Open*.

101. Bhatt DL, Szarek M, Steg PG, et al. Sotagliflozin in Patients with Diabetes and Recent Worsening Heart Failure. N Engl J Med. Jan 14 2021;384(2):117-128. doi:10.1056/NEJMoa2030183

102. Mullens W, Dauw J, Martens P, et al. Acetazolamide in Acute Decompensated Heart Failure with Volume Overload. N Engl J Med. Aug 27 2022;doi:10.1056/NEJMoa2203094

103. Solomon SD, McMurray JJV, Claggett B, et al. Dapagliflozin in Heart Failure with Mildly Reduced or Preserved Ejection Fraction. N Engl J Med. Aug 27 2022;doi:10.1056/NEJMoa2206286

104. Kalra PR, Cleland JGF, Petrie MC, et al. Intravenous ferric derisomaltose in patients with heart failure and iron deficiency in the UK (IRONMAN): an investigator-initiated, prospective, randomised, open-label, blinded-endpoint trial. Lancet. Dec 17 2022;400(10369):2199-2209. doi:10.1016/S0140-6736(22)02083-9

105. Perera D, Clayton T, O'Kane PD, et al. Percutaneous Revascularization for Ischemic Left Ventricular Dysfunction. N Engl J Med. Aug 27 2022;doi:10.1056/NEJMoa2206606

106. Mebazaa A, Davison B, Chioncel O, et al. Safety, tolerability and efficacy of up-titration of guideline-directed medical therapies for acute heart failure (STRONG-HF): a multinational, open-label, randomised, trial. Lancet. Dec 3 2022;400(10367):1938-1952. doi:10.1016/S0140-6736(22)02076-1

107. Sorajja P, Whisenant B, Hamid N, et al. Transcatheter Repair for Patients with Tricuspid Regurgitation. N Engl J Med. Mar 4 2023;doi:10.1056/NEJMoa2300525

108. Lee DS, Straus SE, Farkouh ME, et al. Trial of an Intervention to Improve Acute Heart Failure Outcomes. N Engl J Med. Jan 5 2023;388(1):22-32. doi:10.1056/NEJMoa2211680

109. Mentz RJ, Garg J, Rockhold FW, et al. Ferric Carboxymaltose in Heart Failure with Iron Deficiency. N Engl J Med. Sep 14 2023;389(11):975-986. doi:10.1056/NEJMoa2304968

© 2024 Sayed A et al. *JAMA Network Open*.

110. Brugts JJ, Radhoe SP, Clephas PRD, et al. Remote haemodynamic monitoring of pulmonary artery pressures in patients with chronic heart failure (MONITOR-HF): a randomised clinical trial. Lancet. Jun 24 2023;401(10394):2113-2123. doi:10.1016/S0140-6736(23)00923-6

111. Solomon SD, McMurray JJV, Vaduganathan M, et al. Finerenone in Heart Failure with Mildly Reduced or Preserved Ejection Fraction. New England Journal of Medicine. 2024/09 2024;doi:10.1056/nejmoa2407107

112. Baldus S, Doenst T, Pfister R, et al. Transcatheter Repair versus Mitral-Valve Surgery for Secondary Mitral Regurgitation. New England Journal of Medicine. 2024/08/31 2024;doi:10.1056/nejmoa2408739

113. Anker SD, Friede T, von Bardeleben R-S, et al. Transcatheter Valve Repair in Heart Failure with Moderate to Severe Mitral Regurgitation. New England Journal of Medicine. 2024/08/31 2024;doi:10.1056/nejmoa2314328

114. <Ariel Alonso, Theophile Bigirumurame, Tomasz Burzykowski... - Applied Surrogate Endpoint Evaluation Methods with SAS and R-CRC (2017).pdf>.

115. van Houwelingen HC, Arends LR, Stijnen T. Advanced methods in meta-analysis: multivariate approach and meta-regression. *Stat Med.* 2002;21:589-624.

116. Heerspink HJL, Greene T, Tighiouart H, et al. Change in albuminuria as a surrogate endpoint for progression of kidney disease: a meta-analysis of treatment effects in randomised clinical trials. *Lancet Diabetes Endocrinol.* 2019;7:128-139.

© 2024 Sayed A et al. *JAMA Network Open*.
