# Supplementary material for: Heart Failure and All-Cause Hospitalizations in Patients With Heart Failure: A Meta-Analysis
Source: JAMA Netw Open. 2024 Nov 27;7(11):e2446684. doi: 10.1001/jamanetworkopen.2024.46684 (PMC12549144; doi:10.1001/jamanetworkopen.2024.46684)
Supplement: Supplement 2. — Data Sharing Statement [file jamanetwopen-e2446684-s002.pdf]

Data Sharing Statement

Sayed. Heart Failure and All-Cause Hospitalizations in Patients With Heart Failure. *JAMA Netw Open*. Published November 26, 2024. doi:10.1001/jamanetworkopen.2024.46684

Data
Data available: Yes
Data types: Data (not involving human participants)
How to access data: All of the data and code for this analysis has been uploaded to GitHub to aid in the independent verification and replication of our analysis (https://github.com/ahmedsayedcardio/HF-All-cause-hospitalization-in-HF-trials).
When available: With publication

Supporting Documents
Document types: Statistical/analytic code
How to access documents: All of the data and code for this analysis has been uploaded to GitHub to aid in the independent verification and replication of our analysis (https://github.com/ahmedsayedcardio/HF-All-cause-hospitalization-in-HF-trials).
When available: With publication

Additional Information
Who can access the data: The data and code are available to everyone.
Types of analyses: The data and code are available for any purposes.
Mechanisms of data availability: Any inquiries regarding the data or related statistical code can be directed to Ahmed Sayed (asu.ahmed.sayed@gmail.com)
